# Effects of larval crowding on quantitative variation for development time and viability in *Drosophila melanogaster*

**DOI:** 10.1002/ece3.2552

**Published:** 2016-10-28

**Authors:** Barbara Horváth, Alex T. Kalinka

**Affiliations:** ^1^ Institut für PopulationsgenetikVeterinärmedizinische Universität WienA‐1210ViennaAustria; ^2^ Vienna Graduate School of Population Genetics, Veterinärmedizinische Universität WienA‐1210, ViennaAustria

**Keywords:** development time, *Drosophila melanogaster*, genotype‐by‐environment interaction, larval density, viability

## Abstract

Competition between individuals belonging to the same species is a universal feature of natural populations and is the process underpinning organismal adaptation. Despite its importance, still comparatively little is known about the genetic variation responsible for competitive traits. Here, we measured the phenotypic variation and quantitative genetics parameters for two fitness‐related traits—egg‐to‐adult viability and development time—across a panel of *Drosophila* strains under varying larval densities. Both traits exhibited substantial genetic variation at all larval densities, as well as significant genotype‐by‐environment interactions (GEIs). GEI was attributable to changes in the rank order of reaction norms for both traits, and additionally to differences in the between‐line variance for development time. The coefficient of genetic variation increased under stress conditions for development time, while it was higher at both high and low densities for viability. While development time also correlated negatively with fitness at high larval densities—meaning that fast developers have high fitness—there was no correlation with fitness at low density. This result suggests that GEI may be a common feature of fitness‐related genetic variation and, further, that trait values under noncompetitive conditions could be poor indicators of individual fitness. The latter point could have significant implications for animal and plant breeding programs, as well as for conservation genetics.

## 1 Introduction

The potential for a population to cope with environmental change is contingent upon the maintenance of additive genetic variation for fitness. Although natural selection is expected to erode such variation, several studies have shown that there is abundant genetic variation for fitness‐related traits in natural populations (Fanara, Folguera, Iriarte, Mensch, & Hasson, 2006; Ledón‐Rettig, Pfennig, & Crespi, 2010; McGuigan, Nishimura, Currey, Hurwit, & Cresko, 2010; Paaby et al., 2015; Telonis‐Scott, McIntyre, & Wayne, 2005). This puzzling fact has been explained various ways (e.g., Barton & Turelli, 1989). A potential explanation lies in the highly polygenic nature of life‐history traits; it creates the opportunity for ample amount of nonadditive genetic variation—dominance, epistatic, and genotype‐by‐environment interactions (GEIs)—to exist. Recently, there have been studies quantifying dominance effects and epistasis (Armbruster, Bradshaw, & Holzapfel, 1997; Huang et al., 2012; Monnahan & Kelly, 2015), as well as the role of GEI in adaptation (e.g., Gutteling, Riksen, Bakker, & Kammenga, 2007; Jarosz & Lindquist, 2010; Rohner et al., 2013; for a review see Schlichting, 2008). Despite that, the pervasiveness of additive‐ and nonadditive variation—particularly for competitive traits—is still not yet well understood, especially in the context of environmental changes.

It has been proposed that environmental perturbations can increase both phenotypic and additive genetic variance for fitness traits (Parsons, 1987). To test this hypothesis and to study how changing environmental conditions can affect traits, empirical studies used several abiotic environmental factors to sensitize phenotypes. Among these, there is heat shock (Bateman, 1959; Berger, Bauerfeind, Blanckenhorn, & Schäfer, 2011; Rendel, 1959; Rutherford & Lindquist, 1998; Suzuki & Nijhout, 2006; Waddington, 1953), chemical agents, such as Hsp90 inhibitors (Jarosz & Lindquist, 2010; Queitsch, Sangster, & Lindquist, 2002; Rohner et al., 2013) or ether vapor (Gibson & Hogness, 1996; Waddington, 1956). Less extreme stresses have also been shown to increase phenotypic variation, such as altered salinity levels for the expression of body size variation in sticklebacks (McGuigan et al., 2010). Although mainly morphological traits were initially surveyed, some studies looked at the environmental sensitivity of fitness and fitness‐related traits too (Fowler, Semple, Barton, & Partridge, 1997; Gardner, Fowler, Barton, & Partridge, 2005; Paaby et al., 2014).

Among temperature, salinity, and other abiotic factors, natural populations are also influenced by biotic variables, such as density. Yet, the way of how these biotic factors affect phenotypic traits has received far less attention. Larval density varies greatly in natural *Drosophila* populations, resulting in continuously changing density‐dependent selection (Leips & Mackay, 2000), and affecting life span as well as other fitness‐related traits, such as egg‐to‐adult development time (DT) and body size (Barker & Podger, 1970; Graves & Mueller, 1993; Miller & Thomas, 1958; Prout & McChesney, 1985). Studies found that heat‐shock resistance, as a physiological trait, was insensitive of density (Bubliy, Imasheva, & Loeschcke, 1998), but phenotypic variation for morphological traits increased when exposed to high density (Imasheva & Bubliy, 2003). It has also been shown that larval density is a key factor in successfully selecting for altered life span, suggesting that high density can activate adaptive variation for a fitness‐related trait (Clare & Luckinbill, 1985). Here, we studied whether larval density can similarly affect the expression of phenotypic variation for two further fitness‐related traits, DT, and egg‐to‐adult viability (EAV).

DT is a complex trait, which correlates with many life‐history traits, such as adult body weight‐ and size, age‐specific fecundity or viability (Chippindale, Alipaz, Chen, & Rose, 1997; Chippindale, Alipaz, & Rose, 2004; Nunney, 1996; Prasad, Shakara, Anitha, Rajamani, & Joshi, 2001; Zwaan, Bijlsma, & Hoekstra, 1995). DT is also important ecologically, especially in *Drosophila* species in which the larvae typically live in ephemeral environments experiencing strong con‐specific competition (Throckmorton, 1975), and hence is expected to have a major impact on fitness. As a result, DT is considered to be subject to strong directional selection, which would consequently reduce genetic variation (Nunney, 1996). Nevertheless, fly populations harbor considerable genetic variation for DT when exposed to selection (Cortese, Norry, Piccinali, & Hasson, 2002; Fanara et al., 2006; Neyfakh & Hartl, 1993; Nunney, 1996) or when developmental genes are disrupted (Mensch et al., 2008). In this latter study, Mensch et al. (2008) also found ample amount of GEI for DT when the temperature was manipulated, suggesting that the *Drosophila* genome has a large potential to respond to environmental factors. EAV has also been the subject of numerous studies, which provided valuable information on the genetics and environmental sensitivity of this trait. In *Drosophila melanogaster*, the genetic variation underpinning EAV can be shaped by both mutation–selection balance and diversifying selection in different populations (Mukai & Nagano, 1983; Tachida, Matsuda, Kusakabe, & Mukai, 1983; Tachida & Mukai, 1985; Takano, Kusakabe, & Mukai, 1987). Moreover, it has also been suggested that loci underlying EAV are located primarily in noncoding regions of the genome (Takano et al., 1987). Studies of cactophilic *Drosophila* showed that polymorphic inversions also play an important role in the maintenance of variation for viability (Betrán, Santos, & Ruiz, 1998; Fernandez Iriarte & Hasson, 2000; Rodriguez, Fanara, & Hasson, 1999).

Here, we measured the phenotypic and quantitative genetic variation for DT and EAV among 31 wild‐derived inbred *Drosophila* strains in several competitive conditions: under low, medium, and high larval density treatments. A central aim of the study was to explore how a dynamic component of the biotic environment affects the expression of major life‐history and fitness‐related traits. We demonstrated that GEI is a common characteristic of the two measured fitness traits, appearing as both rank‐order change among the lines and increased phenotypic variance. We then looked at how the evolutionary potential of the traits—in the form of expressed genetic variation—changed along the environmental gradient. We show that DT and EAV respond differently to density stress, with DT showing increasing adaptive potential with the increased level of stress.

## 2 Materials and Methods

### 2.1 *Drosophila melanogaster* fly strains

We measured the phenotypic response of 31 DGRP strains (*D. melanogaster* Genetic Reference Panel, Mackay et al., 2012), which are inbred strains derived from a wild North American population with completely sequenced genomes. The DGRP strains are characterized by high genetic (Mackay et al., 2012; Massouras et al., 2012) and phenotypic variation (Ayroles et al., 2009; Durham, Magwire, Stone, & Leips, 2014; Ellis et al., 2014; Harbison, McCoy, & Mackay, 2013; Mackay et al., 2012; Unckless, Rottschaefer, & Lazzaro, 2015). Fly stocks were ordered from the Bloomington Stock Center and are kept in standard molasses/soy‐corn flour/agar media‐containing vials. They were maintained at 18°C in the dark and transferred to a fresh vial every 2 weeks. Each strain was propagated in 10 vials.

### 2.2 Experimental populations

**Figure 1:**
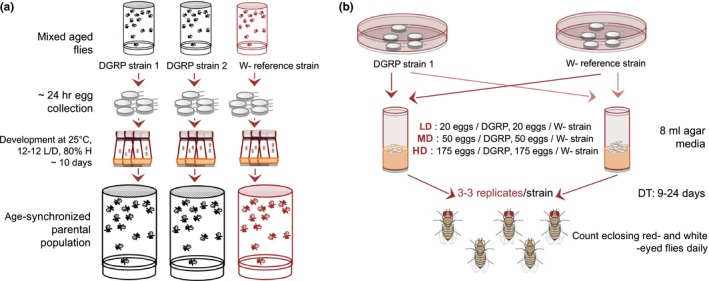
Experimental scheme and setup. (a) To avoid age effects of the parental assay population, we set up 10 synchronously developing bottles for every DGRP strain, individually containing 50 ml standard agar media and ~500 eggs in each bottle. On the 11th day post‐egg transfer, 1,500–2,000 age‐synchronized flies were used to set up the assay populations. (b) We used embryos from an hour‐long egg collection on each day to set up replicates of the three treatments

Flies were kept on standard molasses/soy‐corn flour/agar media‐containing bottles and were maintained at 25°C, 80% humidity, and a 12‐hr:12‐hr light:dark schedule starting from 10 a.m. to 10 p.m. Prior to the start of the experiment, we reared flies under the above conditions for at least three generations to acclimatize them. To minimize variation associated with parental age and rearing conditions, the parents of flies used in this experiment were age‐synchronized by collecting eggs within a 24‐hr window and were grown at low density (10–15 flies/ml food) (Figure 1a). At 0–2 days posteclosion, 1,500–2,000 flies were used from each DGRP strain to set up large egg‐laying cages (ø 100 mm, one cage/DGRP strain). We let the flies acclimatize for 2 days in the cages prior to the start of the experimental egg collections, while we fed them twice a day with fresh yeast paste, which was spread on 2.5% blackcurrant agar plates. We used blackcurrant juice for plate preparation instead of apple juice, because the dark pink color provides a better contrast for the subsequent egg counting (Figure 1b).

### 2.3 Egg collection

On the first experimental day, caged flies were fed at 10 a.m. with a small amount of fresh yeast, and then were placed into carton boxes to allow egg laying in dark conditions, resulting in a better egg yield. Eggs collected on a fresh plate within the first hour (from 4 to 5 p.m.) were discarded because females tend to retain fertilized eggs in their reproductive tracts. To produce synchronized embryos (Neyfakh & Hartl, 1993), we used eggs from the subsequent 1‐hr collection (from 5 to 6 p.m.). Plates were removed from the cages and embryos were carefully washed with water into egg collection chambers. Immediately after this, embryos were transferred back to the agar plates and were counted and sorted with a fine brush under a stereoscope. We repeated the assay three times on consecutive days, yielding three replicate measurements per strain.

### 2.4 Density treatments

To get an estimate of the egg‐to‐adult competitive ability (DT and EAV) of these flies, we caged equal numbers of eggs from each strain and from a reference white‐eye strain (w^1118^) together under three different conditions—one in which the ratio of eggs to food was sufficient to support development of all the larvae (low‐density treatment, 20–20 eggs/vial, 40 in total), one in which the majority of larvae could complete their development (medium‐density treatment, 50–50 eggs/vial, 100 in total) and one in which the ratio of eggs to food supports a smaller fraction of adult eclosion (high‐density treatment, 175–175 eggs/vial, 350 in total) (Figure 1). The white‐eye strain served as a standardized intergenotype competitor in each experimental vial. These treatments, with the additional reference genotype, provide an ecologically relevant measurement of fitness (Joshi, 2001; Joshi & Mueller, 1996). All the experiments used 20‐mm vials containing approximately 8 ml of the above‐mentioned standard media. The number of white‐ and red‐eyed, freshly emerged adults was recorded daily at 2 p.m. from the ninth day on, until no more flies eclosed. EAV was estimated as the proportion of emerging adults relative to the initial number of eggs seeded in each of the experimental vials (20, 50, or 175). DT was measured as the time elapsed from the day of egg collection until the emergence of the adult flies, and it was scored separately for males and females. We recorded DT for every fly until no more flies eclosed and used the mean DT across all the eclosed flies in a vial as the DT estimate for each replicate in each strain.

### 2.5 Statistical analysis

Generalized linear mixed‐effects models (GLMM, R package “lme4,” Bates, Maechler, Bolker, & Walker, 2015) were used to determine which variables explain variation in DT and EAV. These models allow for analyzing non‐normal, heterogeneous nested data with repeated measures (Zuur, Ieno, Walker, Saveliev, & Smith, 2009). We modeled DT as a Poisson‐distributed variable and used log as the link function in the models. For EAV, we utilized the binomial distribution with a logit link function. The following initial model was fitted to DT: *Y*_*ijklm*_ *= *μ *+ L*_*i*_ *+ D*_*j*_ *+ S*_*k*_ *+ V*_*l*_ *+ L*_*i*_ *D*_*j*_ *+ L*_*i*_ *S*_*k*_ *+ D*_*j*_ *S*_*k*_ *+ L*_*i*_ *D*_*j*_ *S*_*k*_ *+ *ε_*ijklm*_, where μ is the overall mean, *L*_*i*_ is the fixed effect of inbred strain, *D*_*j*_ is the fixed effect of density (low, medium, or high), *S*_*k*_ is the fixed effect of sex, *V*_*l*_ is the random effect of the replicate vial (to account for the nonindependence among data points) and ε_*ijklm*_ represents the error term. We fitted this model to a dataset that contained the DT of individual flies instead of replicate means, and thus, the residual variance corresponded to individual measurement errors. To test the effects of the above‐mentioned parameters, we proceeded with dropping terms starting with the three‐way interaction, to find the minimal model that explains the data best. For comparing models with and without the predictors, we used chi‐square tests on the model AIC values (Akaike's information criterion; Crawley, 2007; Zuur et al., 2009; Field, Miles, & Field, 2012). For EAV, our trait of interest was the proportion of eggs that survived to adulthood. To analyze this data, we added a matrix to our data frame containing the number of survivors in each replicate vial in the first column (successes) and the number of nonsurvivors in the second column (failures). The fitted binomial GLMM for EAV did not include the sex effect because the sex of the eggs was unknown: *Y*_*ijk*_ *= *μ *+ L*_*i*_ *+ D*_*j*_ *+ L*_*i*_ *D*_*j*_ *+ *ε_*ijk*_. A significant strain effect indicates genetic differences between the inbred strains for the trait of interest, while a significant effect of density indicates phenotypic plasticity. The interaction terms test whether inbred strains (*L*_*i*_ *D*_*j*_) or males and females (*D*_*j*_ *S*_*k*_) differ in their response to the changing environmental conditions, and indicate a genotype‐by‐environment interaction (GEI) if significant (Fanara et al., 2006).

We also used reduced linear mixed models to partition sources of variance and to estimate heritability for each trait and for each density treatment separately. The following model was fitted to the EAV data for all the three densities: *Y*_*ij*_ *= *μ *+ L*_*i*_ *+ *ε_*ij*,_ where μ is the mean, *L*_*i*_ tests the random effect of inbred strain (*i *=* *1–31) and ε_*ij*_ is the error term. For the log‐transformed DT data, the model included the fix effect of sex and the random effect of replicate vial as well: *Y*_*ijkl*_ *= *μ *+ L*_*i*_ *+ S*_*j*_ *+ L*_*i*_ *S*_*j*_ *+ V*_*k*_ *+ *ε_*ijkl*_. Broad‐sense heritability (repeatability) was calculated as *H* ^2^ *=* $\sigma_{L}^{2}/(\sigma_{L}^{2}+\sigma_{\mathrm{LS}}^{2}+\sigma_{V}^{2}+\sigma_{\mathrm{residual}}^{2}$) for DT (Ober et al., 2012), and as 4*$\sigma_{L}^{2}/(\sigma_{L}^{2}$  *+ *π^2^/3) for EAV (Davies, Scarpino, Pongwarin, Scott, & Matz, 2015), where $\sigma_{L}^{2}$ is the variance of the random line effect and π^2^/3 is the variance of the logistic distribution. To eliminate the scale effects and therefore have a comparable measure of variability of the traits across the densities, we used coefficients of variation (CVs; Houle, 1992). We calculated the phenotypic coefficient of variation as CV_P_ *= *100√($\sigma_{P}^{2}$/*X)*, where $\sigma_{P}^{2}$ is the phenotypic variance (standard deviation of the mean, *SD*) and *X* is the population mean. Coefficient of genetic variation was calculated as CV_G_ *= *100*√[($\sigma_{P}^{2}$*H ^2^)/*X*], where *H* ^2^ is the broad‐sense heritability estimate.

A significant GEI can be the result of either changing between‐line variances or change in the rank order of genotypes (phenotypic plasticity; Fanara et al., 2006). To get a measure for the former one, and to test for inhomogeneity of variances across low, medium, and high densities, we used Levene's test, applying the robust Brown–Forsythe variant of the test (Brown & Forsythe, 1974), which uses deviations from median values instead of means. The test was applied to untransformed DT and EAV values using the following formulae (Dworkin, 2005): *l*_*iD*_ *= |y*_*iD*_ *− y*_*D*_ *|*, where *l*_*i*_ refers to the Levene statistic for the *i* ^*th*^ DGRP strain, y_*i*_ is the median phenotype of the *i* ^*th*^ strain (across replicates), *y* is the population median, and subscript *D* indicates the density treatment (low, medium, or high density). Levene's statistic tests the null hypothesis that there is no heterogeneity among the variances at high, medium, and low densities. To test for rank‐order changes across the three densities, we subtracted line‐mean ordered genotype IDs for both DT and EAV, ranked them by using low‐density data as a reference, and calculated the Kendall's coefficient of concordance (Kendall's *W*; Friedman, 1937). We also examined correlations between DT, EAV, and other traits that have been measured in the DGRP by other research groups (e.g., Ayroles et al., 2009; Durham et al., 2014; Ellis et al., 2014; Harbison et al., 2013; Unckless et al., 2015). All correlation coefficients reported in the results are Spearman's ρ. Statistical analyses were carried out in R (version 3.1.2, R Development Core Team, 2016).

## 3 Results

### 3.1 Larval density affects mean DT and EAV

We scored a total of 24,028 flies derived from 48,050 eggs for EAV and DT under low (40 larvae, LD), medium (100 larvae, MD), and high (350 larvae, HD) larval densities. Overall mean EAV was 49.5%, which declined at high density (EAV_LD_ *= *56.2 ± 23% (±*SD*), EAV_MD_ *= *56.1 ± 18%, and EAV_HD_ *= *36.6 ± 16%). Mean DT increased with increasing larval densities (DT_LD_ *= *10.13 ± 0.58 days, DT_MD_ *= *10.8 ± 0.94 days, and DT_HD_ *= *14.38 ± 2.85 days [Tables 1 and S1]).

**Table 1:** Summary statistics for development time (DT) and viability (EAV)

Trait	Sex	Density:	LD (20–20)	MD (50–50)	HD (175–175)
Development time	M	Mean	10.185	10.832	14.358
		*SD*	0.537	0.979	2.822
		CV_P_ (%)	22.957	30.068	44.333
		CV_G_ (%)	10.670	15.529	16.833
		*H* ^2^	0.216	0.267	0.144
		*N*	804	1,256	2,847
	F	Mean	10.078	10.722	14.631
		*SD*	0.621	0.905	2.876
		CV_P_ (%)	24.827	29.056	44.332
		CV_G_ (%)	11.710	16.245	17.492
		*H* ^2^	0.222	0.313	0.156
		*N*	881	1,381	3,110
Viability	Merged	Mean	0.562	0.561	0.366
		*SD*	0.23	0.18	0.16
		CV_P_ (%)	63.380	55.676	65.062
		CV_G_ (%)	56.884	41.131	53.478
		*H* ^2^	0.806	0.546	0.645
		*N*	1,685	2,637	5,957

*SD* refers to the phenotypic standard deviation; CV_P_ is the phenotypic coefficient of variation, calculated as CV_P_ = 100√($\sigma_{P}^{2}$/*X*), CV_G_ is the genetic coefficient of variation, calculated as CV_G_ = 100√($\sigma_{P}^{2}$*H ^2^/*X*). *N* is the number of flies. DT is in days, and viability is given as proportion of survivals. Individual fly values were used for DT, while the replicate averages for EAV.

### 3.2 Substantial genetic variation and G × E interactions for DT

**Figure 2:**
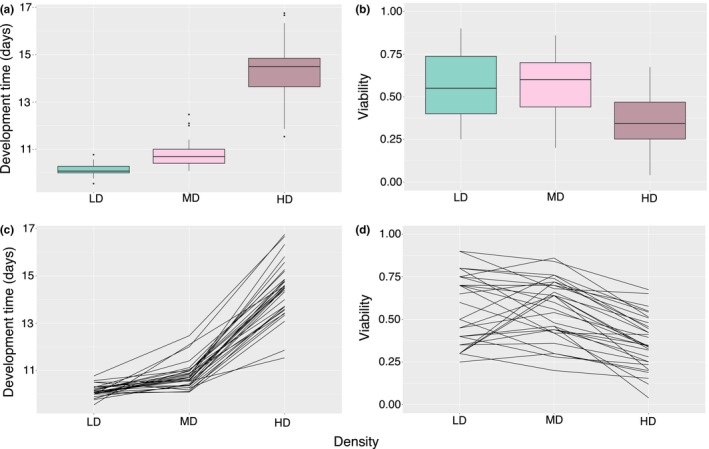
Between‐strain variation for DT (a, c) and EAV (b, d) at low, medium, and high larval densities. (a) and (b) show the overall variation with medians taken across the replicates for each strain (five replicates for low, three replicates for medium and high densities). (c) and (d) illustrate changes in the reaction norms of the 31 DGRP strains using median strain values (sexes pooled for EAV). The nonparallel reaction norms indicate genotype‐environment interactions. LD, low density; MD, medium density; HD, high density; M, males; F, females

**Figure 3:**
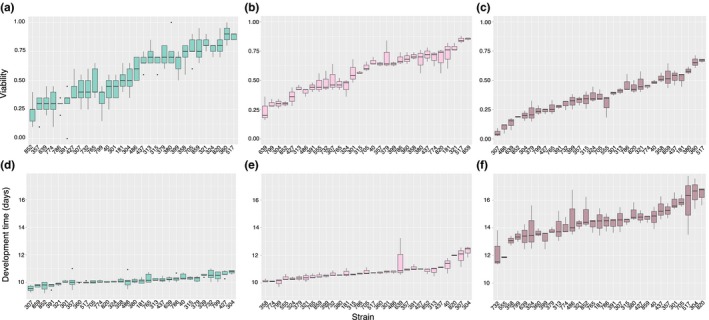
Phenotypic variation for DT and EAV across 31 DGRP strains showing values for five or three replicates in each strain and ranking strains by median phenotype values. (a) EAV, low‐density treatment. (b) EAV, medium‐density treatment. (c) EAV, high‐density treatment. (d) DT at low density, (e) DT at medium density, (f) DT at high density. LD, low density; MD, medium density; HD, high density

To test for possible genotype‐by‐environment interactions, we fitted GLMMs for both DT and EAV data. Prior to model fitting, we pruned the data and retained only complete observations (i.e., where data were available for a strain at all three densities, and at least one observation was recorded for both males and females). *p* Values were obtained by chi‐square tests on the model AIC values, where models with and without the effect in question were compared. For DT, we found that the minimal adequate model included two of the three possible two‐way interaction terms, indicating that GEI is a common, important feature of DT. The three‐way interaction term including sex, genotype, and density effects was dropped as being nonsignificant (*p *>* *.05), and so was the genotype‐by‐sex interaction (*p *>* *.05). The genotype‐by‐density interaction was highly significant, indicated by both the chi‐square test (ΔAIC* = *7, *p *=* *1.25E‐06; Table 2) and the crossover reaction norms in Figure 2, with 7.6% of the genetic variance being due to this interaction effect. Moreover, we obtained a significant sex‐by‐density interaction for DT (ΔAIC* = *3, *p *=* *.027), whereby females developed faster under low‐density conditions and slower at high‐density [mean±*SE*: HD: 14.36 ± 0.11 (M) vs. 14.63 ± 0.17 days (F); MD: 10.83 ± 0.13 (M) vs. 10.72 ± 0.18 days (F); LD: 10.19 ± 0.12 (M) vs. 10.08 ± 0.15 days (F)]. We performed model fitting on the mean‐centered DT data as well, but the results remained unchanged, and thus, we report results on the original data. By visually inspecting it, the model residuals did not deviate from a normal distribution. Effect sizes were calculated on the untransformed DT values. Density expectedly had a major effect on DT (ΔAIC* = *730, *p *=* *2.95E‐40; Table 2), increasing it from low density to medium by 0.66 days (±0.18, *SE*), and by 4.48 days (±0.17) when density was high. Similarly, the strain effect was also significant itself (ΔAIC* = *100, *p *=* *1.66E‐22) (Table 2, Figures 2 and 3).

**Table 2:** GLMM analysis of DT and EAV to test for significant genotype‐by‐environment interactions

Trait	Variable	Δ AIC	*p* Value
Development time	Sex × density × genotype	−106	1
	Sex × genotype	−23	.2124
	Sex × density	3	.0274*
	Density × genotype	7	1.25E‐06***
	Sex	2	.0542
	Genotype	100	1.66E‐22***
	Density	730	2.95E‐40***
	Vial	−2	1
Viability	Density × genotype	157.8	1.01E‐33***
	Genotype	59.1	1.53E‐12***
	Density	62.1	4.52E‐15***

To test for significant GEI, we step by step removed interaction terms from a saturated model and retained variables if they led to a significant increase in AIC value. Δ AIC indicates the change in AIC value when a particular interaction term is removed from the model. To get significance for the main effects, we compared a model with all the main effects and the model lacking the particular variable. We retained the random experimental vial effect in every model in order to utilize the same mixed‐effects models for the comparisons.

*p <* *.05, ***p < .001.

### 3.3 Sex‐by‐density interaction for DT

We obtained a significant sex‐by‐density interaction for DT, whereby females completed their development faster in low‐density conditions, but were slower compared with males when density was high. To further test this interaction, we set up experiments with additional lower and higher densities for three chosen DGRP lines, to have a higher resolution of where the shift in DT occurs. Among the three lines, RAL 301 and RAL 307 followed the observed pattern of females being faster at low density in the original DT dataset, while the pattern for line RAL 555 was not so clear. We used densities of 10–10 (D1), 20–20 (D2), 50–50 (D3), 100–100 (D4), 175–175 (D5), and 250–250 flies (D6) [DGRP line *+ *w^1118^ reference competitor] and again measured DT the same way as above. To test for significant GEI, we fitted an identical GLMM model for these data as described above for DT. We found that females were again faster in low experimental densities [e.g., D1: 9.59 ± 0.67 (M) vs. 9.46 ± 0.56 days (F); Table 3, Figure S1). However, the models did not reveal a significant sex‐by‐density interaction (ΔAIC = −7, *p *>* *.1), despite the observation that above the density D3 (100–100 flies) females tended to slow down in two of the three assayed lines (Table 3, Figure S1). The difference between mean DT of males and females at the highest density for line 301 was as substantial as 2.12 days (~50 hr; Figure S4). Additionally, we found a significant genotype‐by‐sex interaction (ΔAIC* = *2, *p *=* *.0395), and a highly significant genotype‐by‐density effect as well (ΔAIC* = *22, *p *=* *7E‐06). The reason for not finding the significant sex‐by‐density effect might be the combination of high interindividual variation in DT tested for only a few lines, as shown in Figure S1.

**Table 3:** Summary statistics for the density experiments

Trait	Sex	Density	D1	D2	D3	D4	D5	D6
Development time	M	Mean	9.594	9.956	10.688	11.803	14.382	16.795
		*SD*	0.665	0.633	0.788	1.265	2.379	3.331
	F	Mean	9.457	9.730	10.679	11.925	14.433	17.527
		*SD*	0.561	0.574	0.771	1.319	2.389	3.397
Viability	Merged	Mean	0.744	0.728	0.653	0.597	0.523	0.366
		*SD*	0.224	0.177	0.131	0.086	0.095	0.162

Table 3 shows averages for the three DGRP lines studied (RAL 301, 307, and 555). *SD* refers to the phenotypic standard deviation, and the studied densities were as follows: 10–10 (D1), 20–20 (D2), 50–50 (D3), 100–100 (D4), 175–175 (D5), and 250–250 flies (D6) [DGRP line *+ *w^1118^ reference competitor].

### 3.4 Strong density‐by‐strain interaction for EAV

Chi‐square statistics on deviances of the binomial GLMMs showed that including both density (ΔAIC* = *62.1, *p *=* *4.52E‐15) and genotype effect (ΔAIC* = *59.1, *p *=* *1.53E‐12) produced a significant improvement in the model fit for EAV. Fitting the genotype‐by‐density interaction term substantially decreased the residual variance, suggesting that the GEI term was also significant (ΔAIC* = *157.8, *p *=* *1.01E‐33). In addition, we obtained the lowest AIC value for this full model. The genetic variance explained by the genotype‐by‐density interaction was 18.9% (Table 2).

### 3.5 Test for rank‐order changes and inhomogeneous variances as signs of GEI

We showed above that both DT and EAV expressed strong genotype‐by‐environment interactions (Tables 2 and 4). A significant GEI can be the result of either change in the rank order of lines in the different environments, or by change in the among‐line variances (Fanara et al., 2006). To test for inhomogeneity of variances across the three treatments, we applied Levene's test to both EAV and DT. DT showed significantly higher variance at both high (*F*_1,59_ = 20.09, *p *=* *3.46E‐05) and medium (*F*_1,59_ = 5.84, *p *=* *.0186) densities when compared to low density. The contrast remained for DT when comparing high density with medium density (*F*_1,60_ = 8.58, *p *=* *.0048; Figure 2a,c). For EAV, the effect of larval density was not as apparent; EAV showed no difference in variance between low‐ and medium‐density (*F*_1,59_ = 2.59, *p *=* *.113; Figure 2b,d) or medium‐ and high‐density treatments (*F*_1,60_ = 1.12, *p *=* *.295). However, the variance was significantly elevated in the low‐density treatment when compared to the high‐density treatment (*F*_1,59_ = 7.68, *p *=* *.007).

**Table 4:** Reduced mixed‐effects models for DT, providing estimates of variance components and heritability

Trait	Density	Variable	Δ AIC	*p* Value	σ^2^ (%)
Development time	LD	Sex × genotype	−24.9	2.089E‐07***	7.6
		Sex	−11.5	2.458E‐04***	Fixed
		Genotype	−68.0	6.173E‐17***	**14.7**
		Vial	−6.7	.0032**	3.7
		Residuals			74.0
	MD	Sex × genotype	−2.9	.027*	1.2
		Sex	−13.0	1.096E‐04***	Fixed
		Genotype	−81.0	8.42E‐20***	**28.4**
		Vial	−5.9	.0048**	1.8
		Residuals			68.6
	HD	Sex × genotype	−26.2	1.106E‐07***	1.5
		Sex	−20.3	2.426E‐06***	Fixed
		Genotype	−62.1	1.171E‐15***	**11.4**
		Vial	−30.5	1.211E‐08***	1.6
		Residuals			85.5

Models were run on log‐transformed DT data. To estimate the effects of variables, we compared model AIC values with and without the variable in question. Variance components (σ^2^) were calculated from the full models fitting genotype, genotype‐by‐sex and replicate vial as random effects. Broad‐sense heritabilities can be calculated as variance due to genotype divided by the full variance (Ober et al., 2012) and are marked in the table with bold numbers. *p < .05, **p < .01, ***p < .001.

We used the Levene's test to further test whether the within‐strain interindividual variance was greater at high density than at low or medium density for DT. After applying a Bonferroni correction, 30 of 31 strains showed significantly greater variance between individuals at high vs. low, as well as high density vs. medium density for DT (Figures S2 and S3, Table S2; no equivalent test can be applied to EAV as each estimate is an aggregate over all of the individuals that eclose). We also found significant differences in variance for 10/30 lines between the two lower densities (Figures S2 and S3, Table S2). Greater interindividual variance at high density for DT indicates that high larval density elicits developmental noise in the rate of development of an individual larva (Willmore & Hallgrimsson, 2005).

These results are consistent with an increase in genetic variation in stressful conditions, but there is an alternative explanation. Greater developmental noise within strains could contribute to random, nongenetic differences between strains for DT, possibly producing the same signature. This does not appear to be the case here, however. At high density, the between‐strain variance is significantly greater than interindividual variance (Kruskal–Wallis test; χ^2^ *= *926 (30 *df*), *p *=* *2E‐175; Figure S2), suggesting that between‐strain, genetic variance is the major contributor to the signature of GEI. Furthermore, if the significant GEI is due to noise, then we expect DT measures at high density to show more variance, but be uncorrelated with those at lower densities. Instead, there is a significant positive correlation between the strain means at medium‐ and high‐density treatments for DT (ρ = 0.60, *p *=* *3.1E‐04), indicating that DT measures at high density are genetic properties of the strain, rather than due to increased developmental noise. On the other hand, we found no correlation between low and medium densities for DT (ρ = −0.05, *p *=* *.78), and similarly, no correlation between low and high densities (ρ = −0.22, *p *=* *.25) indicating that the low‐density treatment is not stressful. A correlation between medium‐ and high‐density treatments suggests that the medium‐density treatment is also stressful (100 larvae in total), although not as stressful as the high‐density treatment: The standard deviation of hatching times at medium density (σ = 0.56 days) is only half that at high density (σ = 1.19 days). In contrast, there is a nearly fivefold difference between low and high densities (low density: σ = 0.26 days). Such a dramatic increase in hatching delay is likely to have significant consequences in natural populations.

To test whether the observed GEI results from rank‐order changes among the strains, we calculated Kendall's *W* for all the three possible comparisons (LD vs. MD, LD vs. HD, and MD vs. HD), for both DT and EAV data. The correspondence in rank orders was generally low for both traits in every comparison—we did not obtain any significant correlation, suggesting that substantial changes occurred in the rankings of the studied lines (see also Figure 2). For DT, we found the least agreement in rank orders between low and high densities (*W* = 0.167, *p *>* *.1), followed by the low‐density–medium‐density comparison (*W* = 0.197, *p *>* *.1). The highest agreement was found for the medium and high densities (*W* = 0.256, *p *>* *.1). For EAV, the correspondence was generally higher than for DT, and all comparisons resulted in similar values (LD‐MD: *W* = 0.337, *p *>* *.1; LD‐HD: *W* = 0.346, *p *>* *.1; MD‐HD: *W* = 0.337, *p *>* *.1).

### 3.6 Coefficients of variation and heritability for DT and EAV

We utilized reduced models to infer quantitative genetics parameters for both DT and EAV. These models revealed significant genotype, sex, and genotype‐by‐sex effects for DT (Table 4), with broad‐sense heritability being similar for low and high densities ($H_{\mathrm{LD}}^{2}$ = 14.7%; $H_{\mathrm{HD}}^{2}$ = 11.4%), but increased when larval density was medium ($H_{\mathrm{MD}}^{2}$ = 28.4%). The highest genotype‐by‐sex contribution was observed for low density (7.6%), followed by high (1.5%) and medium densities (1.2%). Broad‐sense heritability estimates were generally higher for EAV than for DT, but the estimates showed a different pattern: Highest heritability was estimated for low density ($H_{\mathrm{LD}}^{2}$ = 0.806), while both medium and high densities showed intermediate heritability values ($H_{\mathrm{MD}}^{2}$  = 0.546, $H_{\mathrm{HD}}^{2}$ = 0.645).

**Figure 4:**
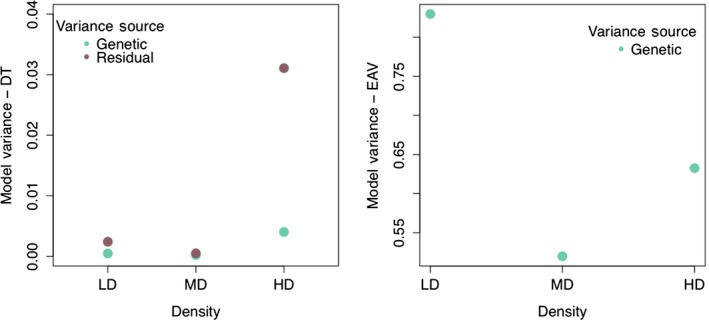
Line and residual variances from the reduced models for DT. Estimated variance components were subtracted from the reduced DT and EAV models to compare changes in genetic and environmental variances across densities. Genetic/residual variance proportions for DT were 0.198 for low density, 0.414 for medium density, and 0.129 for high density

Phenotypic (CV_P_) and genetic (CV_G_) coefficients of variation were also calculated for DT and EAV by using variances, trait mean values, and estimated heritability across the three environments in order to predict whether the evolutionary potential of the traits change with the stress level. CVs provided us with unbiased variance estimates correcting for the link between trait means and variances (scaling effect). For DT, we found that CV_P_ was lowest for low density, and gradually increased to high density (CV_P‐LD_ = 24.03%; CV_P‐MD_ = 29.58%; CV_P‐HD_ = 44.36%; Table 1). We observed the same trend regardless if we used interindividual trait measure variation in the equation (as reported above), or included only single median trait values in the coefficient calculation (CV_P‐LD_ = 16.12%; CV_P‐MD_ = 22.84%; CV_P‐HD_ = 28.76%), or even if we used replicate information (CV_P‐LD_ = 20.06%; CV_P‐MD_ = 24.49%; CV_P‐HD_ = 30.99%). Similarly, we calculated the genetic coefficients of variation (CV_G_) using the heritability estimates from the reduced models. We showed that CV_G_ was lowest for low density and increased to high density (CV_G‐LD_ = 9.18%; CV_G‐MD_ = 15.68%; CV_G‐HD_ = 16.42%). This result is an independent confirmation of the above‐described increase in genetic variation. Moreover, we found this despite the increase in environmental variance along with the genetic variances (Figure 4), suggesting that density as a stress factor can activate adaptive variation for a fitness‐related trait and have a pronounced effect on evolvability.

For EAV, we observed a different pattern: CV_P_ was the lowest under medium‐density conditions and highest at high density (CV_P‐LD_ = 60.29%; CV_P‐MD_ = 55.78%; CV_P‐HD_ = 65.06%). Similar to DT, including not only median measures, but the used three or five replicates per line did not change this trend (CV_P‐LD_ = 63.38%; CV_P‐MD_ = 55.68%; CV_P‐HD_ = 66.58%), suggesting that the quality of measurements for both traits were adequate. CV_G_ values showed high similarity for low and high densities (CV_G‐LD_ = 56.88%; CV_G‐HD_ = 53.48%), and a substantial reduction for medium density (CV_G‐MD_ = 41.13%, Table 1).

### 3.7 Sex ratios

Although we did not include sex in the EAV model, we tested whether there was a deviation from the expected 50:50 sex ratio in any of the three environments. Overall, there were more females eclosing from the experimental vials than males (52.26% [F], 47.74% [M]). This ratio is unchanged when the data are split into low density (52.28% [F], 47.72% [M]), medium density (52.37% [F], 47.63% [M]), and high density (52.21% [F], 47.79% [M]). Not surprisingly, the per‐line replicates were more consistent for medium and high densities (Figure S4). The strongest density effect was observed for line RAL 820, where under high‐density conditions there were four times more females emerging than males (Figure S4).

### 3.8 Correlation with adult‐to‐adult competitive fitness

DT exhibited a significant negative correlation with adult‐to‐adult competitive, reproductive fitness (measured using the competitive index technique in Ayroles et al., 2009) at both high (ρ = −0.56, *p *=* *.0016) and medium densities (ρ = −0.54, *p *=* *.0026), but not at low density (ρ = 0.19, *p *=* *.336), in keeping with the notion that fast egg‐to‐adult development in *Drosophila* is closely related to overall fitness (Throckmorton, 1975), especially under competitive conditions. This result also demonstrates that fitness‐related genetic variation is uncovered by the stressful medium‐ and high‐density treatments. In contrast, there was no relationship between EAV and fitness at high (ρ = 0.26, *p *=* *.16), medium (ρ = 0.17, *p *=* *.36) or low density (ρ = 0.16, *p *=* *.44). In addition, there was no correlation between EAV and DT.

### 3.9 Negative correlation between larval viability and adult body size

Previous studies have found a decrease in larval viability when larger adult body size is artificially selected (Partridge & Fowler, 1993). Similarly, our results revealed a significant negative correlation between EAV and adult body size (data obtained from Durham et al., 2014) under high‐density conditions (ρ = −0.41, *p *=* *.029). To determine whether egg or larval viability was predominantly related to body size, we also measured egg viability for all 31 strains (data not shown). Observations suggested that pupal viabilities do not vary substantially between strains; therefore, we did not account for differences in pupal survival. After correcting for egg viability and contrasting only larval viability with body size, we found that the correlation is predominantly driven by viability of the larvae (larval viability: ρ = −0.49, *p *=* *.0074; egg viability: ρ = −0.09, *p *=* *.63; Figure S5). A relationship between larval viability and adult body size suggests that small larval body size can be advantageous under competitive conditions, perhaps because the larvae of small‐bodied adults need to feed less in food containing metabolic waste products.

## 4 Discussion

Effects of competition and crowding have been widely studied on various aspects of *Drosophila* fitness. Early experiments have demonstrated the importance of frequency‐dependent selection in both intraspecific (Levene, Pavlovsky, & Dobzhansky, 1954, 1958; Lewontin & Matsuo, 1963) and interspecific competition (Barker & Podger, 1970), as well as how density affects the expression of life‐history traits (Barker & Podger, 1970; Graves & Mueller, 1993; Miller & Thomas, 1958; Prout & McChesney, 1985). However, very few studies have assayed natural fly populations—mainly various mutant and inversion‐baring strains have been studied, or experiments were performed with a single isofemale line. By utilizing the DGRP, we could test the effects of competition on naturally occurring genetic variation (Mackay et al., 2012) and draw ecologically relevant conclusions about variation for the studied traits. Moreover, studying a panel of genotypes provided us with the opportunity to test for important evolutionary questions, such as how does evolvability of traits change with the increasing level of density stress? Or how pervasive GEIs are for fitness‐related traits?

To test these questions, we performed thorough phenotyping and found for both DT and EAV that the studied genotypes expressed alternative phenotypes as a function of the environment, resulting in significant GEI. GEI is a key factor in evolvability, as it can maintain variation in natural populations; with GEI being present, the strength and direction of selection might be dramatically different between environments (Zajitschek & Bonduriansky, 2014). Two major forms of GEI are distinguished: significant crossover GEI that result in changes in the rank order of genotypes from one environment to another, and GEI manifested in increased overall expressed variation. The former one is the consequence of some genotypes being superior in one environment but inferior in another (Haldane, 1947), while variance increase is likely due to uncovered adaptive genetic variation. While we found evidence for the latter for DT, only crossover reaction norms were observed for EAV. It is possible that variants in genes underlying essential pathways exhibit deleterious effects that cannot be fully ameliorated by benign environmental conditions. EAV, and viability in general, may be predominantly underpinned by processes that are essential in development and physiology, and this might explain why we observed only rank‐order changes for this trait. The absence of marked changes in variance with increasing densities has been also observed for body weight in *D. melanogaster* and *D. simulans* (Barker & Podger, 1970). This suggests that there is trait‐to‐trait variation in the propensity for expressing GEI with traits involving essential processes being more likely to show phenotypic plasticity without changes in the overall phenotypic variance.

Among the significant GEI, we observed a significant interaction between sex and larval density for DT. At low larval density, females tended to develop faster, while at high larval densities development was faster for males. Miller (1964) showed that *D. melanogaster* females indeed develop somewhat faster when larval density is low (<60), but found no evidence for the opposite when density was high. Further studies have found evidence for faster development of females (Paranjpe, Anitha, Chandrashekaran, Joshi, & Sharma, 2005) and males (Mensch et al., 2008) under differing circumstances, supporting the notion that the sex that develops fastest depends on the environmental context, and presumably also the genetic architecture of the flies. The acquisition of sufficient resources to support egg production is the constraint that most likely influences the DT of females, possibly explaining slower development of females at higher densities. The adult body size of males is known to influence female mate choice with larger males receiving more matings (Friberg & Arnqvist, 2003; Pitnick, 1991), and it is possible that faster male development at higher densities reflects a tension between sexual and natural selection—the opportunity for sexual selection may be stronger in low‐density populations (Coltman et al., 1999). Further studies of development at different densities together with measures of adult body size, mating success, and life‐time fecundity will be needed to determine the underlying causes.

Theoretical studies indicate that temporally fluctuating environments can support the maintenance of genetic variation (Kawecki, 2007; Sasaki & Ellner, 1997; Svardal, Rueffler, & Hermisson, 2011), an idea that was first proposed in a verbal model by Fisher (1930), arguing that the expected continual degradation of a population's environment would ensure that additive genetic variance for fitness would never be exhausted. Furthermore, while the expression of hidden, potentially deleterious, genetic variation when simultaneously encountering a novel environment might act to push a small population to extinction, the expression of GEI in a large population that is experiencing a fluctuating degeneration of their environment is much less likely to do so (Reed, Lowe, Briscoes, & Frankham, 2003). In this sense, we envisage a role for GEI in the long‐term stability of a population when experiencing recurring stresses, such as high density, and not just a role limited to an enhanced potential for adaptation to entirely novel environments. Several studies have found that *Drosophila* populations can readily respond to artificial selection for shorter DT with decreases of up to 40 hr from egg to adult (Burke et al., 2010; Chippindale et al., 1997; Nunney, 1996; Prasad et al., 2001; Zwaan et al., 1995), indicating that abundant additive genetic variance for DT is available in natural populations.

Coefficients of variation (CVs) allow us to compare the evolvability of traits in an unbiased way, correcting for potential interdependence between phenotype means and variances (Houle, 1992). By comparing CVs across different environments, we can predict selection outcomes and therefore choose optimal selection regimes, which may be of a great importance for not only breeders but also in conservation biology. We observed that exposure to high larval density increased both CV_P_ and CV_G_ substantially for DT. This is concordant with the observations of Gebhardt and Stearns (1992); that is, decreasing yeast concentration of fly media increases variation for DT. Developing on poorer food resource potentially stresses flies similar to high density, posing stronger competition on conspecifics for limited resources. On the other hand, EAV showed a different pattern: While both low and high densities were characterized by high CV values, the mildly stressful, medium‐density conditions showed comparably little evolutionary potential.

Although it is clear that the genetic variance expressed under crowded larval conditions has adaptive potential, it is necessary to resolve the presence of genetic variance for DT at low density. Relative to morphological traits, DT is complex and highly polygenic, increasing the number of routes by which the trait could be influenced, and thereby increasing the difficulty confronting any potential canalizing mechanism. Recessive mutations will also be exposed in the inbred strains and are likely to lengthen DT to varying degrees in the different strains (Hollingsworth & Maynard Smith, 1955). In addition, the pooling of sexes could potentially inflate the variance. Although there may be reasons why genetic variance is expressed for DT at low density, the relationship between DT and competitive fitness suggests an alternative approach to the problem. If the variance that is of evolutionary importance is not variance in DT per se, but the variance in DT that contributes to genetic variance in competitive fitness, then this trait—competitive fitness—has no relevant variance expressed in DT at low density. From this perspective, the variance in competitive fitness that is expressed via DT at medium and high densities is cryptic (Dworkin, Palsson, Birdsall, & Gibson, 2003).

An increased variance in DT under high larval density was also reported by Miller (1964) and Barker and Podger (1970) in studies of interspecies competition between *D. melanogaster* and *D. simulans* larvae. However, both results were based on one laboratory strain of *D. melanogaster* derived from a single wild‐caught female without subsequent generations of dedicated sib–sib mating, as was used to produce the DGRP strains used in the present study. Hence, it is possible that the strain used by Miller harbored substantially more genetic variation than individual DGRP strains. For this reason, Miller's result could partially reflect the increased variance case of GEI, despite the use of a single strain. Nonetheless, it is also possible that some of the variation measured by Miller arose from stochastic differences between genetically near‐identical individuals, a phenomenon known as developmental noise (Willmore & Hallgrimsson, 2005). In support of this interpretation, we found evidence for both variance increase and developmental noise in our DT measures.

Miller (1964) proposed that the increased variation in DT is a consequence of some larvae pupating prematurely under conditions of starvation with other larvae feeding for extended periods of time in nutrient‐poor conditions. Such a scenario predicts a positive relationship between the length of development of a larva and its adult body size under conditions of high larval density, a relationship that has been observed when artificially selecting for decreased DT (Nunney, 1996). If true, our results would suggest that the plastic response of premature pupation has a genetic basis with variation for this trait segregating in natural populations. It is also possible, however, that some strains have a higher tolerance of larval waste products perhaps allowing them to more efficiently extract nutrients under conditions of crowding. Experimental evolution of *Drosophila* populations under high‐density larval conditions has resulted in the evolution of larvae with an increased tolerance of urea and ammonia (Betrán et al., 1998; Shakarad et al., 2005; Shiotsugu, Leroi, Yashiro, Rose, & Mueller, 1997), possibly suggesting that strains with greater tolerance of waste products might also have a reduced DT. However, the ability to tolerate metabolic waste might instead incur a cost of decreased efficiency of nutrient extraction (Joshi & Mueller, 1996), thereby leading to either an extended DT or a reduced adult body size, or both. Hence, a relationship between tolerance of crowding and tolerance of waste products does not necessarily predict any relationship between DT and adult body size under high density, although tolerance of waste products and premature pupation response need not be mutually exclusive traits.

Although we found that DT in the context of larval crowding correlates with adult‐to‐adult competitive fitness, there was no such correlation for EAV. This is a counterintuitive result because the viability of any given genotype will determine the chance that this genotype survives to reproduce in the next generation, and, hence, would be expected to impact the fitness of the genotype. However, if there is a trade‐off between EAV and fecundity, this would obscure the relationship between EAV and fitness. Such a trade‐off might arise if EAV is strongly dependent on the mother's investment into the yolk of the egg, and if this investment limits the total number of eggs that she can lay (Einum & Fleming, 2000; Mappes & Koskela, 2007). The existence of such a relationship would imply that a significant fraction of the variation in EAV is a maternal effect (Heath, Fox, & Heath, 1999; Kern, Ackermann, Stearns, & Kawecki, 2007).

After controlling for egg viability, we found a significant negative correlation between larval viability and adult body size. Again this appears to be a counterintuitive result as it might be expected that larvae with the best chance of survival are those that have larger body sizes. However, when competing with other larvae for food and space, those that feed for shorter periods of time, and, thus, have smaller adult body sizes, will also be less affected by the negative consequences of competition. Hence, our result hints at the existence of a trade‐off between viability and body size that is exerted in the preadult stage in *Drosophila* under competitive conditions. A potential trade‐off between viability and body size has been proposed to explain the persistence of small‐bodied species (Blanckenhorn, 2000), and our findings suggest that such a trade‐off might be limited to individual life‐cycle stages.

## Conflict of Interest

None declared.

## References

[ece32552-bib-0001] Armbruster, P. , Bradshaw, W. E. , & Holzapfel, C. M. (1997). Evolution of the genetic architecture underlying fitness in the pitcher‐plant mosquito, *Wyeomyia smithii* . Evolution, 51, 451–458. 10.1111/j.1558-5646.1997.tb02432.x 28565340

[ece32552-bib-0002] Ayroles, J. F. , Carbone, M. A. , Stone, E. A. , Jordan, K. W. , Lyman, R. F. , Magwire, M. M. , … Mackay, T. F. C . (2009). Systems genetics of complex traits in *Drosophila melanogaster* . Nature Genetics, 41, 299–307. 19234471 10.1038/ng.332 PMC2752214

[ece32552-bib-0003] Barker, J. S. F. , & Podger, R. N. (1970). Interspecific competition between *Drosophila melanogaster* and *Drosophila simulans*: Effects of larval density on viability, developmental period and adult body weight. Ecology, 51, 170–189.

[ece32552-bib-0004] Barton, N. H. , & Turelli, M. (1989). Evolutionary quantitative genetics: How little do we know? Annual Review of Genetics, 23, 337–370. 10.1146/annurev.ge.23.120189.002005 2694935

[ece32552-bib-0005] Bateman, K. G. (1959). The genetic assimilation of the dumpy phenocopy. American Naturalist, 56, 341–351.

[ece32552-bib-0006] Bates, D. , Maechler, M. , Bolker, B. , & Walker, S. (2015). Fitting linear mixed‐effects models using lme4. Journal of Statistical Software, 67, 1–48.

[ece32552-bib-0007] Berger, D. , Bauerfeind, S. S. , Blanckenhorn, W. U. , & Schäfer, M. A. (2011). High temperatures reveal cryptic genetic variation in a polymorphic female sperm storage organ. Evolution, 65, 2830–2842. 21967425 10.1111/j.1558-5646.2011.01392.x

[ece32552-bib-0008] Betrán, E. , Santos, M. , & Ruiz, A. (1998). Antagonistic pleiotropic effect of second‐chromosome inversions on body size and early life‐history traits in *Drosophila buzzatii* . Evolution, 52, 144–154. 10.1111/j.1558-5646.1998.tb05147.x 28568158

[ece32552-bib-0009] Blanckenhorn, W. U. (2000). The evolution of body size: What keeps organisms small? Quarterly Review of Biology, 75, 385–407. 11125698 10.1086/393620

[ece32552-bib-0010] Brown, M. B. , & Forsythe, A. B. (1974). Robust tests for equality of variances. Journal of the American Statistical Association, 69, 364–367.

[ece32552-bib-0011] Bubliy, O. A. , Imasheva, A. G. , & Loeschcke, V. (1998). Selection for knockdown resistance to heat in *Drosophila melanogaster* at high and low larval densities. Evolution, 52, 619–625. 10.1111/j.1558-5646.1998.tb01661.x 28568321

[ece32552-bib-0012] Burke, M. K. , Dunham, J. P. , Shahrestani, P. , Thornton, K. R. , Rose, M. R. , & Long, A. D. (2010). Genome‐wide analysis of a long‐term evolution experiment with *Drosophila* . Nature, 467, 587–590. 20844486 10.1038/nature09352

[ece32552-bib-0013] Chippindale, A. K. , Alipaz, J. A. , Chen, H.‐W. , & Rose, M. R. (1997). Experimental evolution of accelerated development in *Drosophila*. 1. Developmental speed and larval survival. Evolution, 51, 1536–1551. 10.1111/j.1558-5646.1997.tb01477.x 28568633

[ece32552-bib-0014] Chippindale, A. K. , Alipaz, J. A. , & Rose, M. R. (2004). Experimental evolution of accelerated development in *Drosophila*. 2. Adult fitness and the fast development syndrome In Rose M. R., Passananti H. B. & Matos M. (Eds.), Methuselah flies: A case study in the evolution of aging (pp. 413–435). Singapore: World Scientific Publishing.

[ece32552-bib-0015] Clare, M. J. , & Luckinbill, L. S. (1985). The effects of gene‐environment interaction on the expression of longevity. Heredity, 55, 19–29. 3930428 10.1038/hdy.1985.67

[ece32552-bib-0016] Coltman, D. W. , Smith, J. A. , Bancroft, D. R. , Pilkington, J. , MacColl, A. D. , Clutton‐Brock, T. H. , & Pemberton, J. M. (1999). Density‐dependent variation in lifetime breeding success and natural and sexual selection in Soay rams. American Naturalist, 154, 730–746. 10.1086/303274 10600616

[ece32552-bib-0017] Cortese, M. D. , Norry, F. M. , Piccinali, R. , & Hasson, E. (2002). Direct and correlated responses to artificial selection on developmental time and wing length in *Drosophila buzzatii* . Evolution, 56, 2541–2547. 12583594 10.1111/j.0014-3820.2002.tb00179.x

[ece32552-bib-0018] Crawley, M. J. (2007). The R book, 1st edn New Jersey: Wiley, ISBN‐13: 9780470510247.

[ece32552-bib-0019] Davies, S. W. , Scarpino, S. V. , Pongwarin, T. , Scott, J. , & Matz, M. V. (2015). Estimating trait heritability in highly fecund species. G3, 5, 2639–2645. 26438295 10.1534/g3.115.020701 PMC4683637

[ece32552-bib-0020] Durham, M. F. , Magwire, M. M. , Stone, E. A. , & Leips, J. (2014). Genome‐wide analysis in *Drosophila* reveals age‐specific effects of SNPs on fitness traits. Nature Communications, 5, Article 4338. 10.1038/ncomms5338 25000897

[ece32552-bib-0021] Dworkin, I. (2005). Canalization, cryptic variation and developmental buffering: A critical examination and analytical perspective. Chapter 8 In Hallgrimsson B. & Hall B. K. (Eds.) Variation: A central concept in biology (pp. 131–158). Waltham, Massachusetts, USA: Academic Press.

[ece32552-bib-0022] Dworkin, I. , Palsson, A. , Birdsall, K. , & Gibson, G. (2003). Evidence that *Egfr* contributes to cryptic genetic variation for photoreceptor determination in natural populations of *Drosophila melanogaster* . Current Biology, 13, 1888–1893. 14588245 10.1016/j.cub.2003.10.001

[ece32552-bib-0023] Einum, S. , & Fleming, I. A. (2000). Highly fecund mothers sacrifice offspring survival to maximize fitness. Nature, 405, 565–567. 10850714 10.1038/35014600

[ece32552-bib-0024] Ellis, L. L. , Huang, W. , Quinn, A. M. , Ahuja, A. , Alfrejd, B. , Gomez, F. E. , et al. (2014). Intrapopulation genome size variation in *D. melanogaster* reflects life history variation and plasticity. PLoS Genetics, 10, e1004522. 25057905 10.1371/journal.pgen.1004522 PMC4109859

[ece32552-bib-0025] Fanara, J. J. , Folguera, G. , Iriarte, P. F. , Mensch, J. , & Hasson, E. (2006). Genotype by environment interactions in viability and developmental time in populations of cactophilic *Drosophila* . Journal of Evolutionary Biology, 19, 900–908. 16674586 10.1111/j.1420-9101.2006.01084.x

[ece32552-bib-0026] Fernandez Iriarte, P. , & Hasson, E. (2000). The role of the use of different host plants in the maintenance of the inversion polymorphism in the cactophilic *Drosophila buzzatii* . Evolution, 54, 1295–1302. 11005296 10.1554/0014-3820(2000)054[1295:trotuo]2.0.co;2

[ece32552-bib-0027] Field, A. , Miles, J. , & Field, Z. (2012). Discovering statistics using R. 1st edn Thousand Oaks, California, USA: Sage Publications, ISBN‐13: 9781446200469.

[ece32552-bib-0028] Fisher, R. (1930). The genetical theory of natural selection. Oxford: Clarendon Press.

[ece32552-bib-0029] Fowler, K. , Semple, C. , Barton, N. H. , & Partridge, L. (1997). Genetic variation for total fitness in *Drosophila melanogaster* . Proceedings of the Royal Society of London B: Biological Sciences, 264, 191–199. 10.1098/rspb.1997.0027 PMC1688253 9061969

[ece32552-bib-0030] Friberg, U. , & Arnqvist, G. (2003). Fitness effects of female mate choice: Preferred males are detrimental for *Drosophila melanogaster* . Journal of Evolutionary Biology, 16, 797–811. 14635895 10.1046/j.1420-9101.2003.00597.x

[ece32552-bib-0031] Friedman, M. (1937). The use of ranks to avoid the assumption of normality implicit in the analysis of variance. Journal of American Statistical Association, 32, 675–701.

[ece32552-bib-0032] Gardner, M. P. , Fowler, K. , Barton, N. H. , & Partridge, L. (2005). Genetic variation for total fitness in *Drosophila melanogaster* . Genetics, 169, 1553–1571. 15545656 10.1534/genetics.104.032367 PMC1449528

[ece32552-bib-0033] Gebhardt, M. D. , & Stearns, S. C. (1992). Phenotypic plasticity for life‐history traits in *Drosophila melanogaster*. III. Effect of the environment on genetic parameters. Genetical Research, 60, 87–101. 1468647 10.1017/s0016672300030780

[ece32552-bib-0034] Gibson, G. , & Hogness, D. S. (1996). Effect of polymorphism in the *Drosophila* regulatory gene *Ultrabithorax* on homeotic stability. Science, 271, 200–203. 8539619 10.1126/science.271.5246.200

[ece32552-bib-0035] Graves, J. L. Jr , & Mueller, L. D. (1993). Population density effects on longevity. Genetica, 91, 99–109. 8125282 10.1007/BF01435991

[ece32552-bib-0036] Gutteling, E. W. , Riksen, J. A. , Bakker, J. , & Kammenga, J. E. (2007). Mapping phenotypic plasticity and genotype‐environment interactions affecting life‐history traits in *Caenorhabditis elegans* . Heredity, 98, 28–37. 16955112 10.1038/sj.hdy.6800894

[ece32552-bib-0037] Haldane, J. B. S. (1947). The interaction of nature and nurture. Eugenics, 13, 197–205. 10.1111/j.1469-1809.1946.tb02358.x 20282564

[ece32552-bib-0038] Harbison, S. T. , McCoy, L. J. , & Mackay, T. F. C. (2013). Genome‐wide association study of sleep in *Drosophila melanogaster* . BMC Genomics, 14, 281. 23617951 10.1186/1471-2164-14-281 PMC3644253

[ece32552-bib-0039] Heath, D. D. , Fox, C. W. , & Heath, J. W. (1999). Maternal effects on offspring size: Variation through early development of chinook salmon. Evolution, 53, 1605–1611. 10.1111/j.1558-5646.1999.tb05424.x 28565562

[ece32552-bib-0040] Hollingsworth, M. J. , & Maynard Smith, J. (1955). The effects of inbreeding on rate of development and on fertility in *Drosophila subobscura* . Journal of Genetics, 53, 295–314.

[ece32552-bib-0041] Houle, D. (1992). Comparing evolvability and variability of quantitative traits. Genetics, 130, 195–204. 1732160 10.1093/genetics/130.1.195 PMC1204793

[ece32552-bib-0042] Huang, W. , Richards, S. , Carbone, M. A. , Zhu, D. , Anholt, R. R. H. , et al. (2012). Epistasis dominates the genetic architecture of *Drosophila* quantitative traits. Proceedings of the National Academy of Sciences of the United States of America, 109, 15553–15559. 22949659 10.1073/pnas.1213423109 PMC3465439

[ece32552-bib-0043] Imasheva, A. G. , & Bubliy, O. A. (2003). Quantitative variation of four morphological traits in *Drosophila melanogaster* under larval crowding. Hereditas, 138, 193–199. 14641483 10.1034/j.1601-5223.2003.01727.x

[ece32552-bib-0044] Jarosz, D. F. , & Lindquist, S. (2010). Hsp90 and environmental stress transform the adaptive value of natural genetic variation. Science, 330, 1820–1824. 21205668 10.1126/science.1195487 PMC3260023

[ece32552-bib-0045] Joshi, A. (2001). Development and competition in *Drosophila*: A tale of two densities. Proceedings of the National Academy of Sciences, India, B67, 389–396.

[ece32552-bib-0046] Joshi, A. , & Mueller, L. D. (1996). Density‐dependent natural selection in *Drosophila*: Trade‐offs between larval food acquisition and utilization. Evolutionary Ecology, 10, 463–474.

[ece32552-bib-0047] Kawecki, T. J. (2007). The evolution of genetic canalization under fluctuating selection. Evolution, 54, 1–12. 10.1111/j.0014-3820.2000.tb00001.x 10937177

[ece32552-bib-0048] Kern, S. , Ackermann, M. , Stearns, S. C. , & Kawecki, T. J. (2007). Decline in offspring viability as a manifestation of aging in *Drosophila melanogaster* . Evolution, 55, 1822–1831. 10.1111/j.0014-3820.2001.tb00831.x 11681737

[ece32552-bib-0049] Ledón‐Rettig, C. C. , Pfennig, D. W. , & Crespi, E. J. (2010). Diet and hormonal manipulation reveal cryptic genetic variation: Implications for the evolution of novel feeding strategies. Proceedings of the Royal Society of London B: Biological Sciences, 277, 3569–3578. 10.1098/rspb.2010.0877 PMC2982244 20573627

[ece32552-bib-0050] Leips, J. , & Mackay, T. F. (2000). Quantitative trait loci for life span in *Drosophila melanogaster*: Interactions with genetic background and larval density. Genetics, 155, 1773–1788. 10924473 10.1093/genetics/155.4.1773 PMC1461186

[ece32552-bib-0051] Levene, H. , Pavlovsky, O. , & Dobzhansky, Th. (1954). Interaction of the adaptive values in polymorphic experimental populations of *Drosophila pseudoobscura* . Evolution, 8, 335–349.

[ece32552-bib-0052] Levene, H. , Pavlovsky, O. , & Dobzhansky, Th. (1958). Dependence of the adaptive values of certain genotypes in *Drosophila pseudoobscura* on the composition of the gene pool. Evolution, 12, 18–23.

[ece32552-bib-0053] Lewontin, R. C. , & Matsuo, Y. (1963). Interaction of genotypes determining viability in *Drosophila busckii* . Proceedings of the National Academy of Sciences of the United States of America, 49, 270–278. 13930343 10.1073/pnas.49.2.270 PMC299796

[ece32552-bib-0054] Mackay, T. F. C. , Richards, S. , Stone, E. A. , Barbadilla, A. , Ayroles, J. F. , Zhu, D. , Casillas, S. , et al. (2012). The *Drosophila melanogaster* genetic reference panel. Nature, 482, 173–178. 22318601 10.1038/nature10811 PMC3683990

[ece32552-bib-0055] Mappes, T. , & Koskela, E. (2007). Genetic basis of the trade‐off between offspring number and quality in the bank vole. Evolution, 58, 645–650. 15119447

[ece32552-bib-0056] Massouras, A. , Waszak, S. M. , Albarca‐Aguilera, M. , Hens, K. , Holcombe, W. , Ayroles, J. F. , et al. (2012). Genomic variation and its impact on gene expression in *Drosophila melanogaster* . PLoS Genetics, 8, e1003055. 23189034 10.1371/journal.pgen.1003055 PMC3499359

[ece32552-bib-0057] McGuigan, K. , Nishimura, N. , Currey, M. , Hurwit, D. , & Cresko, W. A. (2010). Cryptic genetic variation and body size evolution in threespine stickleback. Evolution, 65, 1203–1211. 21463296 10.1111/j.1558-5646.2010.01195.x

[ece32552-bib-0058] Mensch, J. , Lavagnino, N. , Carreira, V. P. , Massaldi, A. , Hasson, E. , & Fanara, J. J. (2008). Identifying candidate genes affecting developmental time in *Drosophila melanogaster*: Pervasive pleiotropy and gene‐by‐environment interaction. BMC Developmental Biology, 8, 78. 18687152 10.1186/1471-213X-8-78 PMC2519079

[ece32552-bib-0059] Miller, R. S. (1964). Interspecies competition in laboratory populations of *Drosophila melanogaster* and *Drosophila simulans* . American Naturalist, 98, 221–238.

[ece32552-bib-0060] Miller, R. S. , & Thomas, J. L. (1958). The effects of larval crowding and body size on the longevity of adult *Drosophila melanogaster* . Ecology, 39, 118–125.

[ece32552-bib-0061] Monnahan, P. J. , & Kelly, J. K. (2015). Epistasis is a major determinant of the additive genetic variance in *Mimulus guttatus* . PLoS Genetics, 11, e1005201. 25946702 10.1371/journal.pgen.1005201 PMC4422649

[ece32552-bib-0062] Mukai, T. , & Nagano, S. (1983). The genetic structure of natural populations of *Drosophila melanogaster*. XVI. Excess of additive genetic variance of viability. Genetics, 105, 115–134. 17246151 10.1093/genetics/105.1.115 PMC1202139

[ece32552-bib-0063] Neyfakh, A. A. , & Hartl, D. A. (1993). Genetic control of the rate of embryonic development: Selection for faster development at elevated temperatures. Evolution, 47, 1625–1631. 10.1111/j.1558-5646.1993.tb02182.x 28564900

[ece32552-bib-0064] Nunney, L. (1996). The response to selection for fast larval development in *Drosophila melanogaster* and its effect on adult weight: An example of a fitness trade‐off. Evolution, 50, 1193–1204. 10.1111/j.1558-5646.1996.tb02360.x 28565282

[ece32552-bib-0065] Ober, U. , Ayroles, J. F. , Stone, E. A. , Richards, S. , Zhu, D. , Gibbs, R. A. , et al. (2012). Using whole‐genome sequence data to predict quantitative trait phenotypes in *Drosophila melanogaster* . PLoS Genetics, 8, e1002685. 22570636 10.1371/journal.pgen.1002685 PMC3342952

[ece32552-bib-0066] Paaby, A. B. , & Rockman, M. V. (2014). Cryptic genetic variation: Evolution's hidden substrate. Nature Reviews Genetics, 15, 247–258. 10.1038/nrg3688 PMC4737706 24614309

[ece32552-bib-0067] Paaby, A. B. , White, A. G. , Riccardi, D. D. , Gunsalus, K. C. , Piano, F. , & Rockman, M. V. (2015). Wild worm embryogenesis harbors ubiquitous polygenic modifier variation. eLife, 4, e09178. doi: 10.7554/eLife.09178 10.7554/eLife.09178 PMC4569889 26297805

[ece32552-bib-0068] Paranjpe, D. A. , Anitha, D. , Chandrashekaran, M. K. , Joshi, A. , & Sharma, V. K. (2005). Possible role of eclosion rhythm in mediating the effects of light‐dark environments on pre‐adult development in *Drosophila melanogaster* . BMC Developmental Biology, 5, 5. 15725348 10.1186/1471-213X-5-5 PMC554107

[ece32552-bib-0069] Parsons, P. A. (1987). Evolutionary rates under environmental stress. Evolutionary Biology, 21, 311–347.

[ece32552-bib-0070] Partridge, L. , & Fowler, K. (1993). Responses and correlated responses to artificial selection on thorax length in *Drosophila melanogaster* . Evolution, 47, 213–226. 10.1111/j.1558-5646.1993.tb01211.x 28568094

[ece32552-bib-0071] Pitnick, S. (1991). Male size influences mate fecundity and remating interval in *Drosophila melanogaster* . Animal Behaviour, 41, 735–745.

[ece32552-bib-0072] Prasad, N. G. , Shakara, M. , Anitha, D. , Rajamani, M. , & Joshi, A. (2001). Correlated responses to selection for faster development and early reproduction in *Drosophila*: The evolution of larval traits. Evolution, 55, 1363–1372. 11525460 10.1111/j.0014-3820.2001.tb00658.x

[ece32552-bib-0073] Prout, T. , & McChesney, F. (1985). Competition among immatures affects their adult fertility: Population dynamics. American Naturalist, 126, 521–558.

[ece32552-bib-0074] Queitsch, C. , Sangster, T. A. , & Lindquist, S. (2002). Hsp90 as a capacitor of phenotypic variation. Nature, 417, 618–624. 12050657 10.1038/nature749

[ece32552-bib-1000] R Development Core Team (2016). R: A language and environment for statistical computing. R Foundation for Statistical Computing, Vienna, Austria http://www.R-project.org.

[ece32552-bib-0075] Reed, D. H. , Lowe, E. H. , Briscoes, D. A. , & Frankham, R. (2003). Fitness and adaptation in a novel environment: Effect of inbreeding, prior environment, and lineage. Evolution, 57, 1822–1828. 14503623 10.1111/j.0014-3820.2003.tb00589.x

[ece32552-bib-0076] Rendel, J. M. (1959). Canalization of the scute phenotype of *Drosophila* . Evolution, 13, 425–439.

[ece32552-bib-0077] Rodriguez, C. , Fanara, J. J. , & Hasson, E. (1999). Inversion polymorphism, longevity, and body size in a natural population of *Drosophila buzzatii* . Evolution, 53, 612–620. 10.1111/j.1558-5646.1999.tb03796.x 28565408

[ece32552-bib-0078] Rohner, N. , Jarosz, D. F. , Kowalko, J. E. , Yoshizawa, M. , Jeffery, W. R. , Borowsky, R. L. , et al. (2013). Cryptic variation in morphological evolution: Hsp90 as a capacitor for loss of eyes in cavefish. Science, 342, 1372–1375. 24337296 10.1126/science.1240276 PMC4004346

[ece32552-bib-0079] Rutherford, S. L. , & Lindquist, S. (1998). Hsp90 as a capacitor for morphological evolution. Nature, 396, 336–342. 9845070 10.1038/24550

[ece32552-bib-0080] Sasaki, A. , & Ellner, S. (1997). Quantitative genetic variance maintained by fluctuating selection with overlapping generations: Variance components and covariances. Evolution, 51, 682–696. 10.1111/j.1558-5646.1997.tb03652.x 28568569

[ece32552-bib-0081] Schlichting, C. D. (2008). Hidden reaction norms, cryptic genetic variation, and evolvability. Annals of the New York Academy of Sciences, 1133, 187–203. 18559822 10.1196/annals.1438.010

[ece32552-bib-0082] Shakarad, M. , Prasad, N. G. , Gokhale, K. , Gadagkar, V. , Rajamani, M. , & Joshi, A. (2005). Faster development does not lead to correlated evolution of greater pre‐adult competitive ability in *Drosophila melanogaster*. 2005. Biology Letters, 1, 91–94. 17148136 10.1098/rsbl.2004.0261 PMC1629059

[ece32552-bib-0083] Shiotsugu, J. , Leroi, A. M. , Yashiro, H. , Rose, M. R. , & Mueller, L. D. (1997). The symmetry of correlated responses in adaptive evolution: An experimental study using *Drosophila* . Evolution, 51, 163–172. 10.1111/j.1558-5646.1997.tb02397.x 28568802

[ece32552-bib-0084] Suzuki, Y. , & Nijhout, H. F. (2006). Evolution of polyphenols by genetic accommodation. Science, 311, 650–652. 16456077 10.1126/science.1118888

[ece32552-bib-0085] Svardal, H. , Rueffler, C. , & Hermisson, J. (2011). Comparing environmental and genetic variance as adaptive response to fluctuating selection. Evolution, 65, 2492–2513. 21884052 10.1111/j.1558-5646.2011.01318.x

[ece32552-bib-0086] Tachida, H. , Matsuda, M. , Kusakabe, S. , & Mukai, T. (1983). Variance component analysis for viability in an isolated population of *Drosophila melanogaster* . Genetical Research, 42, 207–217.

[ece32552-bib-0087] Tachida, H. , & Mukai, T. (1985). The genetic structure of natural populations of *Drosophila melanogaster*. XIX. Genotype‐environment interaction in viability. Genetics, 111, 43–55. 17246297 10.1093/genetics/111.1.43 PMC1202597

[ece32552-bib-0088] Takano, T. , Kusakabe, S. , & Mukai, T. (1987). The genetic structure of natural populations of *Drosophila melanogaster*. XX. Comparison of genotype‐environment interaction in viability between a northern and a southern population. Genetics, 117, 245–254. 3117620 10.1093/genetics/117.2.245 PMC1203201

[ece32552-bib-0089] Telonis‐Scott, M. , McIntyre, L. M. , & Wayne, M. L. (2005). Genetic architecture of two fitness‐related traits in *Drosophila melanogaster*: Ovariole number and thorax length. Genetica, 125, 211–222. 16247693 10.1007/s10709-005-8549-4

[ece32552-bib-0090] Throckmorton, L. H. (1975). The phylogeny, ecology and geography of *Drosophila* . Handbook of Genetics, 3, 421–469.

[ece32552-bib-0091] Unckless, R. L. , Rottschaefer, S. M. , & Lazzaro, B. P. (2015). A genome‐wide association study for nutritional indices in Drosophila. G3, 5, 417–425. 25583649 10.1534/g3.114.016477 PMC4349095

[ece32552-bib-0092] Waddington, C. H. (1953). Genetic assimilation of an acquired character. Evolution, 7, 118–126.

[ece32552-bib-0093] Waddington, C. H. (1956). Genetic assimilation of the bithorax phenotype. Evolution, 10, 1–13.

[ece32552-bib-0094] Willmore, K. E. , & Hallgrimsson, B. (2005). Within individual variation: Developmental noise versus developmental stability. Chapter 10 In Halgrimsson B. & Hall B. K. (Eds.), Variation: A Central Concept in Biology (pp. 191–218). Waltham, Massachusetts, USA: Academic Press.

[ece32552-bib-0095] Zajitschek, F. , & Bonduriansky, R. (2014). Quantitative genetics of wild populations of arthropods. Chapter 9 In Charmantier A., Garant D. & Kruuk L. E. B. (Eds.), Quantitative genetics in the wild (pp. 147–159). Oxford, UK: Oxford University Press.

[ece32552-bib-0096] Zuur, A. F. , Ieno, E. N. , Walker, N. J. , Saveliev, A. A. , & Smith, G. M. (2009). GLMM and GAMM. Chapter 13 In Gail M., Krickeberg K., Samet J., Tsiatis A., & Wong W. (Ed.), Mixed effects models and extensions in ecology with R (pp. 323–339). New York: Springer.

[ece32552-bib-0097] Zwaan, B. , Bijlsma, R. , & Hoekstra, R. F. (1995). Artificial selection for developmental time in *Drosophila melanogaster* in relation to the evolution of aging: Direct and correlated responses. Evolution, 49, 635–648. 10.1111/j.1558-5646.1995.tb02300.x 28565147

