# Supplementary material for: Effects of larval crowding on quantitative variation for development time and viability in Drosophila melanogaster
Source: Ecol Evol. 2016 Oct 28;6(23):8460–73. doi: 10.1002/ece3.2552 (PMC5167028; doi:10.1002/ece3.2552)

**Supplementary materials**

**Table S1.** Median DT and EAV values at low, medium and high larval densities for 31 DGRP strains. We indicate DT for males and females separately (±standard errors, S.E.), while only have a single value for EAV (±S.E.). The last column indicates the number of flies scored for each treatment. LD – low density; MD – medium density; HD – high density. LD measures are based on 5 replicates, while MD and HD measures are based on 3 replicates. *for strain 555 only MD and HD values are measured.

| RAL ID | Density | DT - Male | DT - Female | EAV | N |
| --- | --- | --- | --- | --- | --- |
| 40 | LD | 10.620±0.13 | 10.212±0.17 | 0.420±0.05 | 42 |
| 40 | MD | 11.616±0.40 | 10.964±0.25 | 0.660±0.02 | 99 |
| 40 | HD | 14.742±0.59 | 14.839±0.38 | 0.484±0.01 | 254 |
| 181 | LD | 10.133±0.13 | 10.050±0.11 | 0.510±0.04 | 51 |
| 181 | MD | 10.865±0.10 | 10.62±0.24 | 0.727±0.07 | 109 |
| 181 | HD | 14.574±0.86 | 14.362±0.65 | 0.516±0.04 | 271 |
| 301 | LD | 10.094±0.04 | 9.967±0.13 | 0.440±0.04 | 44 |
| 301 | MD | 10.906±0.09 | 10.69±0.12 | 0.567±0.06 | 85 |
| 301 | HD | 15.289±0.32 | 16.528±0.39 | 0.390±0.01 | 205 |
| 304 | LD | 10.943±0.08 | 10.483±0.07 | 0.510±0.05 | 51 |
| 304 | MD | 12.100±0.56 | 12.363±0.21 | 0.300±0.02 | 45 |
| 304 | HD | 16.544±0.70 | 16.548±0.69 | 0.198±0.02 | 104 |
| 307 | LD | 9.900±0.10 | 9.410±0.09 | 0.410±0.05 | 41 |
| 307 | MD | 12.056±0.53 | 11.847±0.35 | 0.507±0.07 | 76 |
| 307 | HD | 12.833±0.83 | 14.85±0.53 | 0.052±0.02 | 27 |
| 313 | LD | 10.022±0.02 | 10.294±0.05 | 0.720±0.04 | 72 |
| 313 | MD | 10.722±0.25 | 11.059±0.18 | 0.434±0.01 | 65 |
| 313 | HD | 13.824±0.577 | 14.314±0.75 | 0.410±0.01 | 215 |
| 315 | LD | 10.161±0.05 | 10.414±0.11 | 0.660±0.03 | 66 |
| 315 | MD | 10.449±0.05 | 10.805±0.03 | 0.567±0.01 | 85 |
| 315 | HD | 14.211±0.27 | 14.701±0.30 | 0.335±0.04 | 176 |
| 321 | LD | 9.911±0.06 | 9.871±0.05 | 0.820±0.03 | 82 |
| 321 | MD | 10.554±0.31 | 10.236±0.15 | 0.780±0.02 | 117 |
| 321 | HD | 13.916±0.17 | 14.586±0.22 | 0.475±0.05 | 249 |
| 324 | LD | 10.062±0.04 | 10.025±0.03 | 0.770±0.02 | 77 |
| 324 | MD | 10.447±0.08 | 10.155±0.11 | 0.447±0.04 | 67 |
| 324 | HD | 13.772±0.93 | 13.892±0.94 | 0.370±0.04 | 194 |
| 357 | LD | 10.225±0.13 | 9.800±0.34 | 0.270±0.05 | 27 |
| 357 | MD | 11.017±0.19 | 10.936±0.07 | 0.640±0.01 | 96 |
| 357 | HD | 15.094±0.45 | 15.354±0.23 | 0.328±0.02 | 172 |
| 358 | LD | 10.058±0.04 | 10.186±0.11 | 0.720±0.05 | 72 |
| 358 | MD | 10.210±0.15 | 10.089±0.02 | 0.707±0.02 | 106 |
| 358 | HD | 12.832±0.25 | 13.287±0.17 | 0.520±0.02 | 273 |
| 360 | LD | 9.978±0.02 | 10.000±0.00 | 0.900±0.04 | 90 |
| 360 | MD | 10.749±0.09 | 10.708±0.18 | 0.687±0.03 | 103 |
| 360 | HD | 13.426±0.21 | 13.825±0.24 | 0.657±0.03 | 345 |
| 379 | LD | 10.342±0.10 | 10.154±0.14 | 0.710±0.04 | 71 |
| 379 | MD | 10.353±0.18 | 10.326±0.09 | 0.700±0.07 | 105 |
| 379 | HD | 13.867±0.20 | 13.74±0.15 | 0.227±0.05 | 119 |
| 380 | LD | 10.333±0.11 | 9.933±0.13 | 0.750±0.07 | 75 |
| 380 | MD | 10.787±0.04 | 10.267±0.13 | 0.673±0.06 | 101 |
| 380 | HD | 15.861±0.44 | 14.484±0.39 | 0.583±0.02 | 306 |
| 391 | LD | 10.113±0.07 | 9.229±0.16 | 0.290±0.08 | 29 |
| 391 | MD | 11.252±0.16 | 10.931±0.13 | 0.440±0.02 | 66 |
| 391 | HD | 14.207±0.37 | 14.361±0.28 | 0.276±0.01 | 145 |
| 399 | LD | 10.555±0.14 | 10.476±0.07 | 0.650±0.04 | 65 |
| 399 | MD | 10.662±0.12 | 10.391±0.03 | 0.640±0.01 | 96 |
| 399 | HD | 13.145±0.57 | 13.303±0.22 | 0.320±0.04 | 168 |
| 427 | LD | 10.583±0.13 | 10.603±0.19 | 0.420±0.05 | 42 |
| 427 | MD | 11.083±0.08 | 10.864±0.18 | 0.360±0.05 | 54 |
| 427 | HD | 14.765±0.44 | 14.825±0.02 | 0.240±0.01 | 126 |
| 437 | LD | 10.151±0.06 | 10.261±0.11 | 0.660±0.03 | 66 |
| 437 | MD | 11.093±0.05 | 11.149±0.08 | 0.720±0.03 | 108 |
| 437 | HD | 14.360±0.32 | 16.268±0.59 | 0.528±0.04 | 277 |
| 486 | LD | 10.229±0.23 | 9.972±0.25 | 0.600±0.04 | 60 |
| 486 | MD | 10.900±0.06 | 10.636±0.15 | 0.400±0.02 | 60 |
| 486 | HD | 14.508±1.08 | 14.928±1.19 | 0.096±0.02 | 50 |
| 517 | LD | 10.020±0.04 | 9.948±0.03 | 0.880±0.01 | 88 |
| 517 | MD | 10.842±0.08 | 10.659±0.04 | 0.847±0.02 | 127 |
| 517 | HD | 15.957±1.24 | 15.764±1.26 | 0.671±0.01 | 352 |
| 555* | MD | 10.381±0.25 | 10.386±0.08 | 0.467±0.05 | 70 |
| 555* | HD | 11.994±0.10 | 11.816±0.06 | 0.298±0.06 | 156 |
| 639 | LD | 10.183±0.13 | 10.237±0.11 | 0.310±0.04 | 31 |
| 639 | MD | 11.593±0.83 | 11.533±0.86 | 0.240±0.06 | 36 |
| 639 | HD | 13.363±0.49 | 13.427±0.60 | 0.136±0.03 | 71 |
| 705 | LD | 10.157±0.06 | 9.947±0.10 | 0.770±0.05 | 77 |
| 705 | MD | 10.946±0.16 | 10.719±0.18 | 0.614±0.02 | 92 |
| 705 | HD | 15.907±0.48 | 15.903±0.13 | 0.366±0.03 | 192 |
| 732 | LD | 10.803±0.25 | 10.267±0.28 | 0.420±0.06 | 42 |
| 732 | MD | 10.781±0.25 | 10.585±0.04 | 0.480±0.05 | 72 |
| 732 | HD | 12.128±0.98 | 12.33±0.61 | 0.295±0.02 | 155 |
| 765 | LD | 10.100±0.10 | 10.550±0.28 | 0.480±0.06 | 48 |
| 765 | MD | 10.348±0.18 | 10.562±0.14 | 0.467±0.03 | 70 |
| 765 | HD | 13.914±0.29 | 14.545±0.34 | 0.265±0.04 | 139 |
| 774 | LD | 10.200±0.12 | 10.000±0.00 | 0.300±0.06 | 30 |
| 774 | MD | 10.122±0.04 | 10.105±0.03 | 0.693±0.04 | 104 |
| 774 | HD | 13.982±0.39 | 14.161±0.35 | 0.448±0.01 | 235 |
| 786 | LD | 10.093±0.15 | 10.540±0.14 | 0.290±0.02 | 29 |
| 786 | MD | 10.62±0.09 | 10.661±0.09 | 0.680±0.03 | 102 |
| 786 | HD | 13.979±0.44 | 14.784±0.37 | 0.467±0.04 | 245 |
| 799 | LD | 10.500±0.29 | 10.547±0.25 | 0.340±0.05 | 34 |
| 799 | MD | 10.198±0.12 | 9.942±0.22 | 0.300±0.02 | 45 |
| 799 | HD | 13.538±0.34 | 13.139±0.24 | 0.242±0.02 | 127 |
| 820 | LD | 10.050±0.05 | 10.018±0.02 | 0.810±0.04 | 81 |
| 820 | MD | 12.016±0.02 | 11.958±0.08 | 0.687±0.06 | 103 |
| 820 | HD | 16.152±0.44 | 16.706±0.46 | 0.440±0.03 | 231 |
| 852 | LD | 9.889±0.11 | 9.700±0.20 | 0.230±0.05 | 23 |
| 852 | MD | 11.033±0.75 | 10.962±0.12 | 0.300±0.01 | 45 |
| 852 | HD | 14.633±0.49 | 15.136±0.93 | 0.189±0.00 | 99 |
| 859 | LD | 9.708±0.14 | 9.721±0.07 | 0.780±0.05 | 78 |
| 859 | MD | 10.547±0.13 | 10.536±0.09 | 0.854±0.01 | 128 |
| 859 | HD | 14.167±0.41 | 15.125±0.05 | 0.532±0.06 | 279 |

**Table S2.** Levene’s test results (p-values) for 31 DGRP strains testing for developmental noise by comparing inter-individual variances at low, medium and high larval densities. All but one strain (427) showed a significant increase in inter-individual variances between low- and high density after correcting for multiple testing. Similarly, variance further increased for 30/31 strains between medium and high density (exception: 639). In the comparison of low and medium density only 10/31 strains showed significant increase in variance after Bonferroni correction. Test results remaining significant after correcting for multiple testing are shown in bold. N: number of phenotyped flies at a) low density (LD), at b) medium density (MD), and at high density (HD).

| **RAL ID** | **N (LD)** | **N (MD)** | **N (HD)** | **Levene stat (LD-MD)** | **P-value (LD-MD)** | **Levene stat (MD-HD)** | **P-value (MD-HD)** | **Levene stat (LD-HD)** | **P-value (LD-HD)** |
| --- | --- | --- | --- | --- | --- | --- | --- | --- | --- |
| **40** | 42 | 99 | 254 | 3,2648 | 0,07294543 | 34,2376 | **1.12E-08** | 21,9673 | **4,25E-06** |
| **181** | 51 | 109 | 271 | 0,0894 | 0,76531263 | 126,6373 | **1.56E-25** | 62,4384 | **4,47E-14** |
| **301** | 44 | 85 | 205 | 26,5499 | **9,55E-07** | 59,7031 | **1.85E-13** | 57,6814 | **6,35E-13** |
| **304** | 51 | 45 | 104 | 13,7983 | **0,00034526** | 18,6522 | **2.87E-05** | 50,5183 | **4,21E-11** |
| **307** | 41 | 76 | 27 | 8,9796 | 0,00334507 | 29,8375 | **3.39E-07** | 54,8226 | **3,08E-10** |
| **313** | 72 | 65 | 215 | 15,7166 | **0,00011874** | 29,3009 | **1.34E-07** | 54,0521 | **2,08E-12** |
| **315** | 66 | 85 | 176 | 2,5964 | 0,10922464 | 57,5751 | **5.87E-13** | 54,2252 | **2,85E-12** |
| **321** | 82 | 117 | 249 | 6,4099 | 0,01212892 | 47,8938 | **2.04E-11** | 64,0071 | **2,14E-14** |
| **324** | 77 | 67 | 194 | 12,2441 | **0,00062374** | 24,5982 | **1.28E-06** | 38,0365 | **2,54E-09** |
| **357** | 27 | 96 | 172 | 14,3005 | **0,00024352** | 109,1966 | **1.22E-21** | 19,6834 | **1,52E-05** |
| **358** | 72 | 106 | 273 | 0,0995 | 0,75274942 | 49,2506 | **1.05E-11** | 38,6941 | **1,44E-09** |
| **360** | 90 | 103 | 345 | 52,6008 | **9,93E-12** | 50,6599 | **4.42E-12** | 84,3063 | **1,76E-18** |
| **379** | 71 | 105 | 119 | 1,2142 | 0,27201527 | 41,2085 | **8.18E-10** | 33,1967 | **3,35E-08** |
| **380** | 76 | 101 | 306 | 12,5657 | **0,00050405** | 67,4980 | **2.87E-15** | 81,0962 | **1,05E-17** |
| **391** | 29 | 66 | 145 | 2,4055 | 0,12430968 | 55,0271 | **2.93E-12** | 33,8888 | **2,80E-08** |
| **399** | 65 | 96 | 168 | 0,1898 | 0,66368605 | 70,0265 | **3.53E-15** | 50,7296 | **1,33E-11** |
| **427** | 42 | 54 | 126 | 19,8970 | **2,26E-05** | 22,3408 | **4.62E-06** | 6,9549 | 0,0091532 |
| **437** | 66 | 108 | 277 | 2,8390 | 0,0938177 | 103,7826 | **9.99E-22** | 74,9130 | **1,98E-16** |
| **486** | 60 | 60 | 50 | 0,2145 | 0,64408097 | 21,0948 | **1.19E-05** | 19,7449 | **2,15E-05** |
| **517** | 88 | 127 | 352 | 26,8103 | **5,18E-07** | 285,9073 | **1.35E-50** | 265,9439 | **4,60E-47** |
| **555** | 0 | 70 | 156 | - | - | 17,3040 | **4.54E-05** | - | - |
| **639** | 31 | 36 | 71 | 9,7006 | 0,0027398 | 4,9981 | 0.0274919 | 20,3528 | **1,76E-05** |
| **705** | 77 | 92 | 192 | 25,4990 | **1,15E-06** | 79,2243 | **6.91E-17** | 120,6733 | **2,08E-23** |
| **732** | 42 | 72 | 155 | 0,7031 | 0,40351566 | 25,8513 | **7.76E-07** | 18,1073 | **3,24E-05** |
| **765** | 48 | 70 | 139 | 1,0630 | 0,30468088 | 48,9589 | **3.56E-11** | 43,8399 | **3,75E-10** |
| **774** | 30 | 104 | 235 | 0,0003 | 0,9865017 | 54,7790 | **1.09E-12** | 15,9281 | **8,54E-05** |
| **786** | 29 | 102 | 245 | 1,6605 | 0,19983999 | 65,4910 | **1.01E-14** | 23,9929 | **1,66E-06** |
| **799** | 34 | 45 | 127 | 1,7657 | 0,18783570 | 22,0564 | **5.43E-06** | 11,8203 | **0,000748** |
| **820** | 81 | 103 | 231 | 0,3695 | 0,54405819 | 278,4627 | **7.93E-46** | 226,9723 | **7,24E-39** |
| **852** | 23 | 45 | 99 | 10,5712 | 0,00181055 | 34,7080 | **2.66E-08** | 37,3225 | **1,28E-08** |
| **859** | 78 | 128 | 279 | 7,7465 | 0,0058873 | 76,5963 | **5.73E-17** | 74,4316 | **2,13E-16** |

**Figure S1.** Density experiment; we tested differences in DT between males and females across changing larval densities with two selected lines. We competed the two chosen genotypes against a reference line (w^1118^) under 6 different densities. The models did not reveal a significant sex-by-density effect (ΔAIC = -7, *P* > 0.1), although the slowest developing line (RAL 301) shows substantial difference between males and females under high-density conditions. Values show three replicate mean development time values in (A-C). Viability values (D-F) are also shown. G-I shows inter-individual variation for DT.


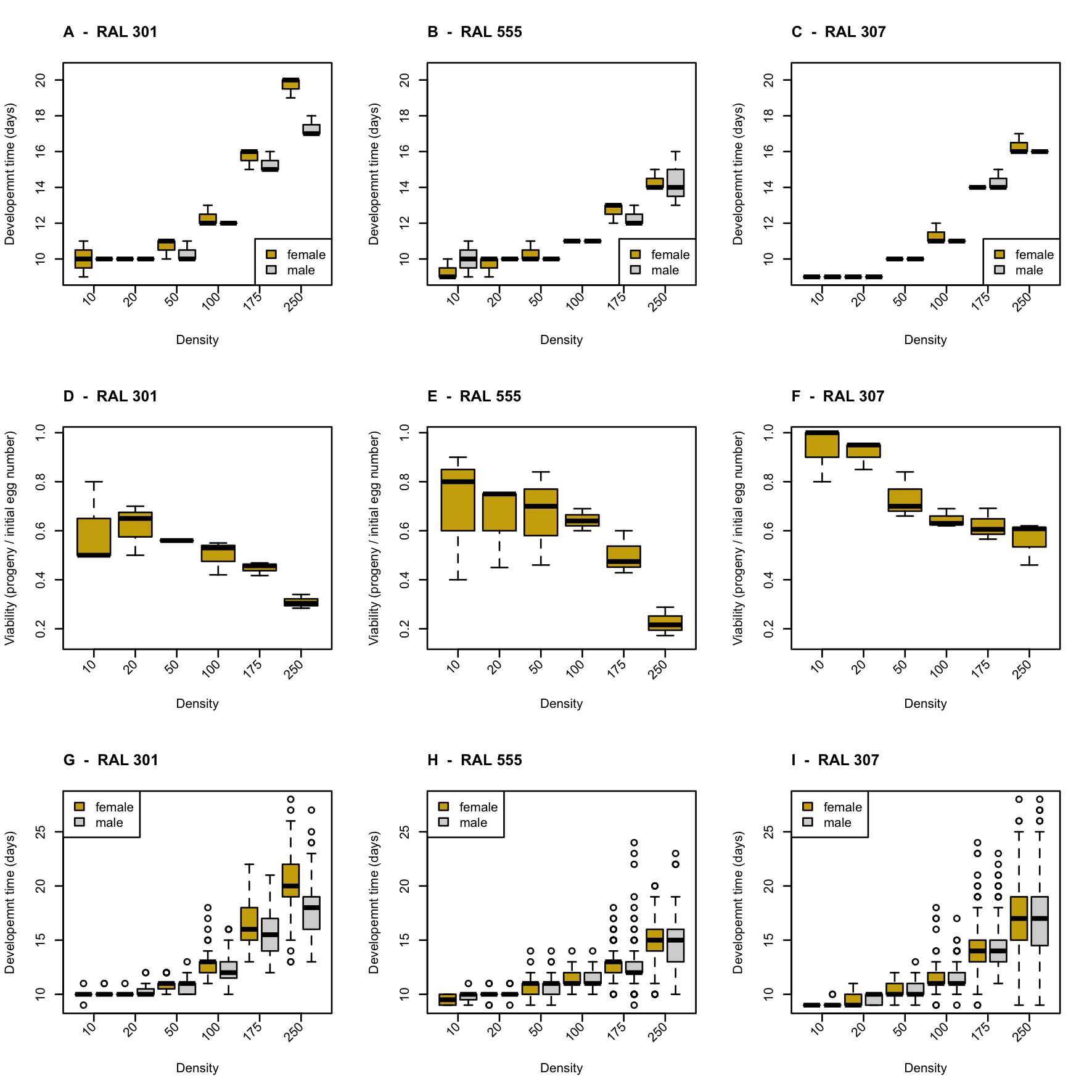
**Figure S2.** Means and standard deviations for DT under high, medium and low larval density (HD, MD and LD, respectively) measured for 31 strains. Low-density measures are based on five replicates, while medium- and high-density measures on three replicates. All but one strain (427) showed a significant increase in inter-individual variances between low- and high density after correcting for multiple testing. Similarly, variance further increased for 30/31 strains between medium and high density (exception: 639). In the comparison of low and medium density only 10/31 strains showed significant increase in variance after Bonferroni correction. Corresponding p-values can be found in Table S2.


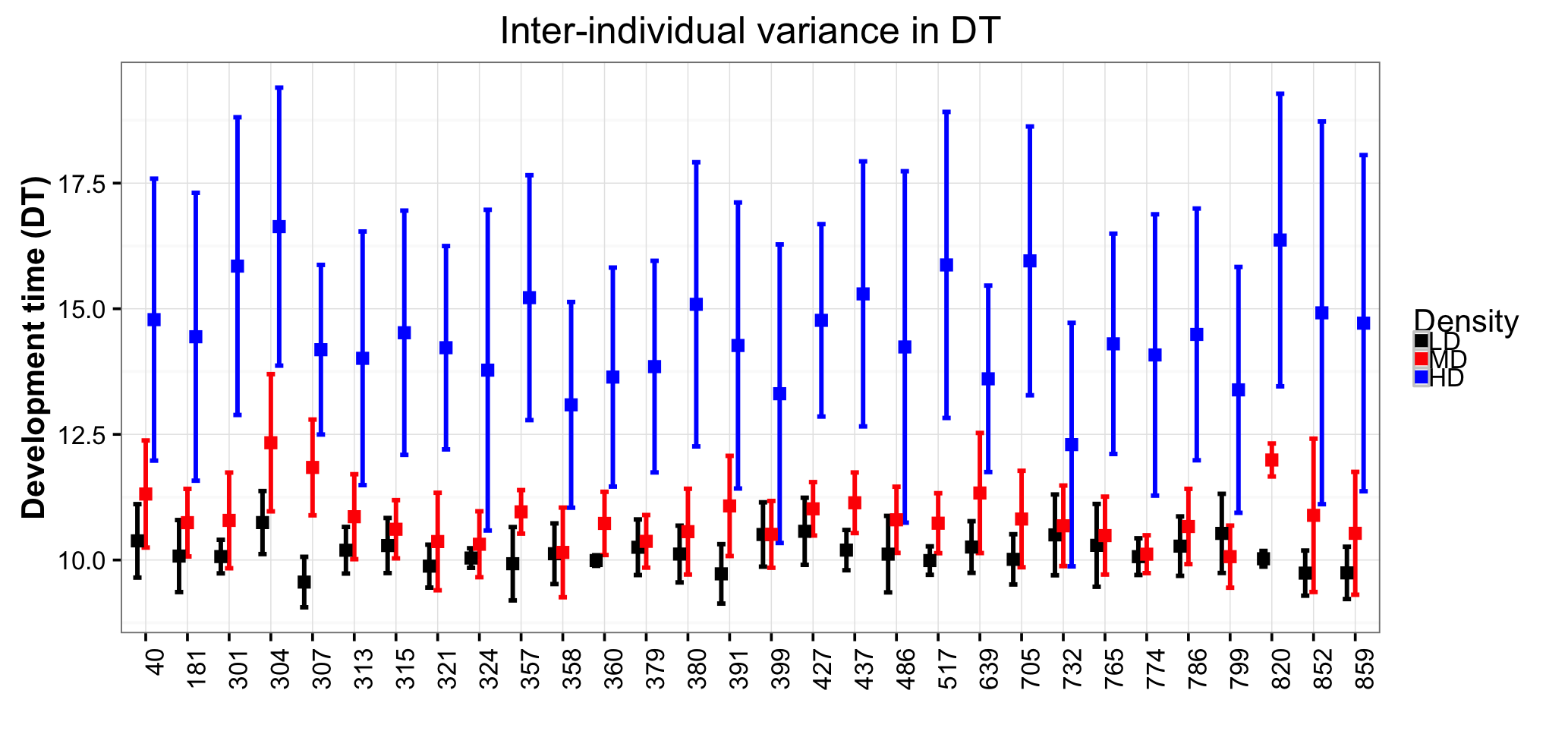


**Figure S3.** Inter-individual variances for DT under low (top panel), medium (middle panel) and high (bottom panel) larval density measured for 31 DGRP strains. A Kruskal-Wallis test (χ^2^ = 926 (30 df), p = 2e-175) indicates that between-strain variance is significantly greater at high density than within-strain, between-individual variance.


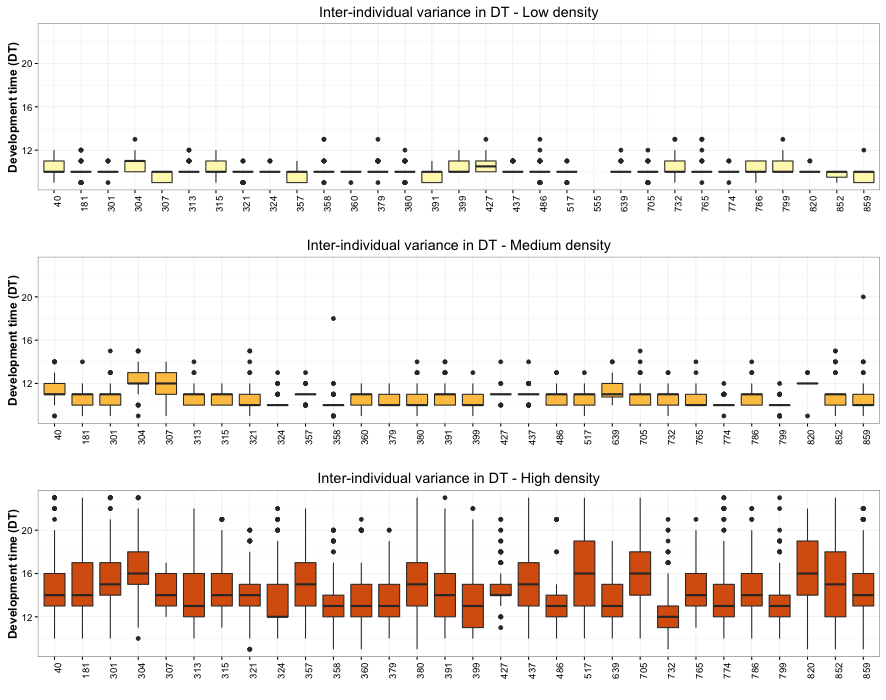


**Figure S4.** Sex ratios at low, medium and high larval densities. Dashed red line indicates the even (50:50) sex ratio, below this the viability of males was lower, while above 1 females showed higher viability.


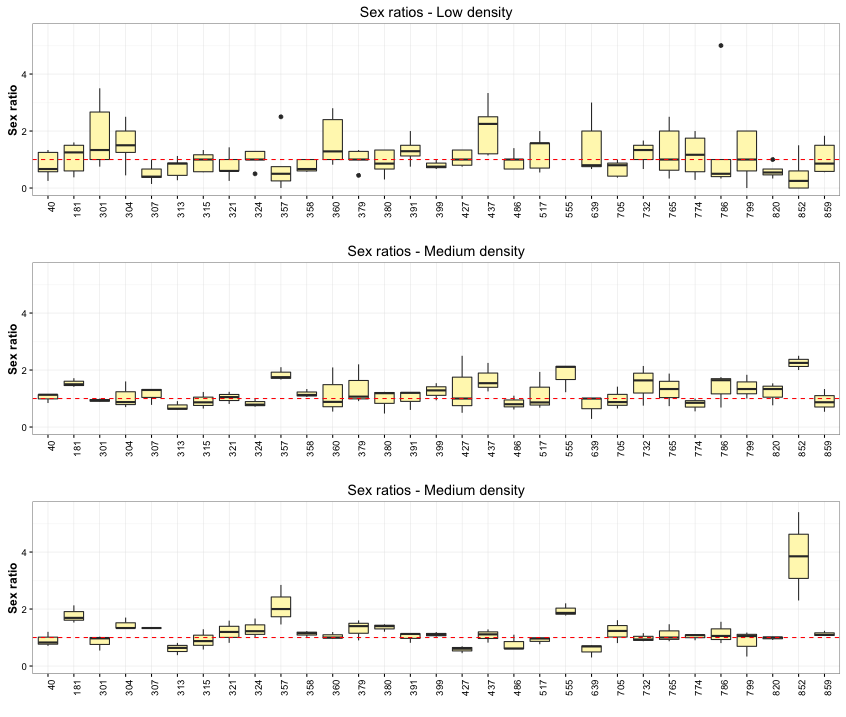


**Figure S5.** Negative relationship between larval viability and body size under low larval density (A), medium density (B) and under high larval density (C). Larval viabilities are taken as the residuals after fitting egg viability to the EAV values. The gradually increasing negative correlation between viability and body size indicates that small body size can be advantageous under competitive, source-limiting conditions. Correlations (A: R^2^ = 0.0136, p = 0.5621; B: R^2^ = 0.134, p = 0.0313; B: R^2^ = 0.232, p = 0.0054) are shown.


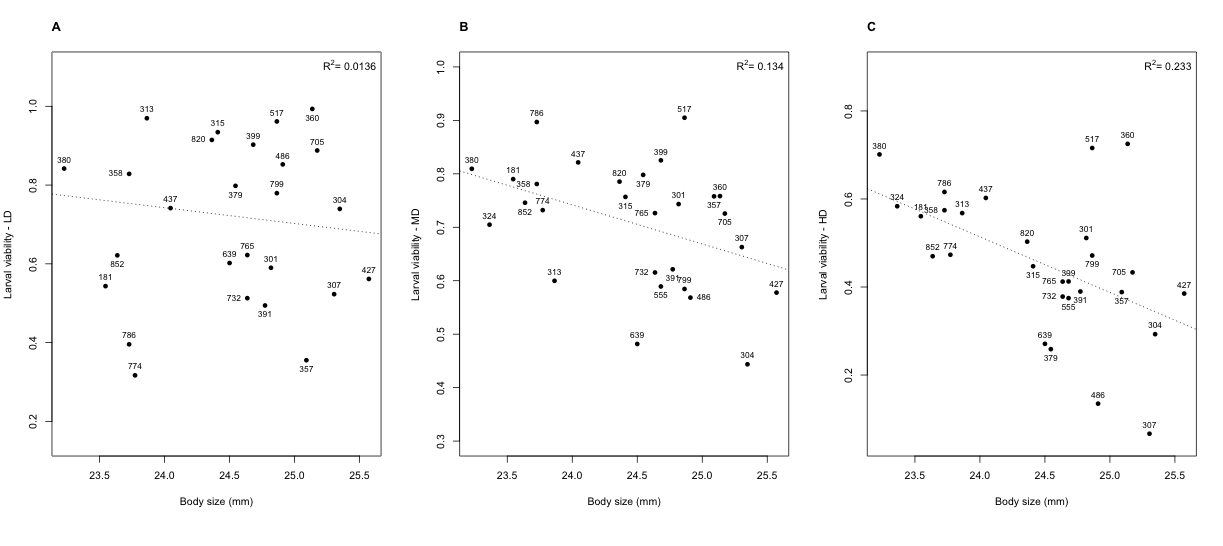

Supplement: Supplementary file 1 [file ECE3-6-8460-s001.docx]
